# An Internet-Based Mindfulness- and Compassion-Based Intercare Program for Reducing Parental Burnout: Randomized Controlled Trial

**DOI:** 10.2196/87416

**Published:** 2026-06-25

**Authors:** Francisco Javier Villalón López, Maximiliano Escaffi-Schwarz

**Affiliations:** 1 Centro de Educación Médica y Simulación Clínica (CEMSIC), Faculty of Medicine, Universidad Diego Portales, avenida Ejercito 141, Santiago, Santiago Metropolitan, Chile, 56 2 2676 2904; 2 Programa de Postgrado en Psiquiatría y Salud Mental, Complejo Asistencial Sótero del Río, Faculty of Medicine, Universidad Diego Portales, Santiago, Santiago Metropolitan, Chile; 3 Facultad de Administración y Economía, Universidad Diego Portales, Santiago, Santiago Metropolitan, Chile

**Keywords:** burnout, parents, psychology, mindfulness, compassion, teleworking, mothers, randomized controlled trials as topic, internet-based intervention, mental health, developing countries, psychological

## Abstract

**Background:**

Parental burnout is an underrecognized syndrome characterized by emotional exhaustion, detachment from children, and reduced parental efficacy. It is associated with sleep disturbance, addictive behaviors, suicidal ideation, and increased risk of child neglect and family conflict. Despite its public health relevance, evidence-based interventions remain limited, particularly in low- and middle-income contexts.

**Objective:**

This study aims to evaluate the efficacy and safety of a mindfulness- and compassion-based intercare program for parental burnout (IBAP-BP)—designed to reduce burnout symptoms in teleworking mothers.

**Methods:**

A 3-arm randomized controlled trial (IBAP-BP, active control, and waitlist) was conducted across Chile (December 2022-March 2023) with a 9-month follow-up. Participants (N=593) were women aged 18 years or older, teleworking ≥1 day/week, and living with at least 1 child. Exclusion criteria were self-reported severe psychiatric disorders. Randomization was computer-generated and centrally concealed; data analysts were blinded. The IBAP-BP group attended 8 weekly 2-hour internet-based sessions plus daily home practice integrating mindfulness and compassion. The active control performed relaxation and reflective journaling matched for duration and structure. The primary outcome was parental burnout at 9 months measured using the average-item score of the Parental Burnout Assessment; secondary outcomes were mindfulness, balance of risks/resources, and adverse effects. Modified intention-to-treat analyses and multilevel structural models assessed effects over time.

**Results:**

Of 593 randomized participants, 343 contributed at least 1 postbaseline assessment and comprised the modified intention-to-treat sample. At 9 months, the prespecified primary analysis showed greater reductions in parental burnout for IBAP-BP compared with the waitlist (mean difference=0.62, 95% CI 0.09-1.14; Cohen *d*≈0.6, 95% CI 0.41-0.77). No significant difference was found between IBAP-BP and the active control, which showed transient improvements up to 3 months. Effects remained robust in sensitivity analyses. Self-reported adverse events were rare and mild across IBAP-BP and active control. Mediation analyses showed inconsistent associations between mindfulness facets and outcomes.

**Conclusions:**

The culturally adapted, internet-based IBAP-BP program was feasible, safe, and effective compared with the waitlist condition for reducing parental burnout in working mothers, with effects sustained over 9 months, although it did not demonstrate superiority over the active control at the primary end point. Internet-based mindfulness- and compassion-based interventions may offer scalable, equitable options for preventing and treating parental burnout in resource-limited settings.

## Introduction

Parental burnout (PB) is a critical yet underrecognized condition, affecting an estimated 0.3% to 8.9% of parents globally. This syndrome, characterized by emotional exhaustion, detachment from children, lack of fulfillment in their role as a parent, and diminished parenting efficacy [1], has profound implications for individual health and family well-being. PB is associated with increased somatic complaints, sleep disturbance, addictive behavior, and suicidal ideation. Furthermore, it has been linked to higher couple conflicts and higher risk of child neglect and violence [2,3]. Therefore, interventions to reduce PB are urgent.

Mindfulness-based interventions (MBIs) have shown promising effects in reducing PB [4-6]. Mindfulness refers to practices and processes that foster present-moment awareness with curiosity and acceptance [7,8], enhancing attention, emotional regulation, and self-regulation, all of which are key mechanisms in stress reduction [7,9]. Providing preliminary support for the effects of mindfulness in reducing PB levels, the Bayot et al [10] trial (n=76) compared a mindfulness–self-compassion program to an active control (AC) and showed reductions in PB in both groups, but with a stronger reduction in the mindfulness–self-compassion group (*η*²=0.45). Furthermore, the 8-week informal mindfulness intervention proposed by Urbanowicz et al [11] (n=30) led to large within-group reductions in PB (*η*²=0.24).

However, there remain important practical and methodological limitations. MBIs are typically time-intensive, which may be challenging for parents already experiencing a high demand-resource imbalance [12,13]. Most studies have been conducted in high-income settings, with limited cultural adaptation and accessibility, particularly in contexts where commuting and economic barriers constrain participation [10,11,14,15]. Finally, in addition to the above practical issues, a Cochrane review of 11 trials (n=2118) found low to very low-certainty evidence for MBIs, stressing the need for further research [6]. Existing trials, including Bayot et al [10] and Urbanowicz et al [11], provide preliminary support but are limited by small samples, short follow-up, and methodological constraints (eg, lack of preregistration or inactive control groups), restricting causal inference.

To address the above issues, we then examine whether an adapted internet-based mindfulness- and compassion-based intercare program (Intercare for Parental Burnout [IBAP]) can reduce PB levels in mothers who telework who are particularly at risk of experiencing PB [2,16]. The IBAP, which was developed in Chile [17], was developed with cultural and modular components, flexible delivery, accelerated instructor training, and an emphasis on belonging through intercare. Prior studies have shown its potential to reduce burnout in physicians during COVID-19 [18] and depressive symptoms in students [19]. IBAP targets key factors linked to PB, including perfectionism, emotion regulation, and social support [13]. To provide a more robust examination of the potential benefits of the IBAP, we implemented a 3-arm randomized controlled trial (RCT) with pre-post measurements and a 9-month follow-up.

The aim of this study is to evaluate the efficacy, feasibility, and safety of an internet-based mindfulness- and compassion-based intercare program for parental burnout (IBAP-BP) in reducing PB among teleworking mothers through a 3-arm RCT (IBAP-BP, active control [AC], and waitlist [WL]). Secondary aims are to examine potential mediators (mindfulness and parental risk-resource balance) and to assess acceptability and adverse effects.

## Methods

### Design

A 3-arm randomized, parallel-group superiority trial with blinded data analysts was conducted in community settings across Chile. Participants were randomly assigned to one of three arms: (1) the mindfulness- and compassion-based intercare program adapted to reduce PB (IBAP-BP), (2) an AC group, and (3) a WL group.

Participant enrollment took place between December 22, 2022, and March 8, 2023. The trial was retrospectively registered at ClinicalTrials.gov (NCT05833269) on April 15, 2023, after enrollment had commenced but prior to the primary outcome assessment period.

Due to an administrative oversight, the third arm was not included in the initial registration and was later added through a registry update on August 15, 2023. All study arms and outcomes had been prespecified in the ethics-approved protocol prior to the start of enrollment, and no primary or secondary outcomes were modified after registration.

A detailed reconciliation of the originally approved design, subsequent amendments, timing of changes, and any protocol deviations is provided in Multimedia Appendix 1 (the *Trial Registration Acknowledgement* section). Reporting adheres to CONSORT (Consolidated Standards of Reporting Trials) (Checklist 1), CONSORT-EHEALTH (Consolidated Standards of Reporting Trials of Electronic and Mobile Health Applications and Online Telehealth), and TIDieR (Template for Intervention Description and Replication) (Checklist 2) guidelines.

### Participants

Eligible participants were women aged 18 years or older, teleworking ≥1 day/week, cohabiting with at least 1 child; criteria reflected funder priorities and prior evidence, suggesting telework as a potential risk context for PB due to work-family boundary blurring and increased role demands [2,16]. There were no treatment restrictions. Exclusion criteria included self-reported severe depression, substance abuse, or psychosis.

Participants were recruited in Chile between December 22, 2022, and March 8, 2023, nationwide through social media advertising and institutional partnerships coordinated by the Chilean Occupational Safety Institute.

### Randomization and Blinding

Participants were centrally randomized by an independent researcher using a computer-generated sequence, with allocation concealed until assignment. Stratification was performed based on baseline PB severity (PBA ≥84), and no blocking was applied.

Due to logistical constraints related to scheduling group sessions, the intervention arms (IBAP-BP and AC) had fixed capacities (n=125 each), whereas the WL group had no capacity restrictions. Randomization was therefore implemented across the full sample, with assignment to intervention arms limited by the available capacity and the remaining participants allocated to the WL group.

Participants were required to confirm their availability before the disclosure of allocation. A subset of participants withdrew prior to the disclosure of allocation and before confirming their participation. These vacant intervention slots were filled with participants from the WL pool, following the original randomization sequence, to ensure the preservation of allocation procedures.

After the 9-month assessment, participants in the AC and WL groups were offered IBAP-BP in an open-label phase. Full randomization and scheduling procedures are detailed in Multimedia Appendix 1.

### Procedure

The IBAP-BP intervention consisted of 8 weekly 2-hour therapist-guided, internet-delivered group sessions led by trained instructors, complemented by 20 to 30 minutes of daily home practice incorporating guided meditation and structured journaling. The program was standardized across 5 cohorts of 25 participants each, with no individual tailoring. Participants received weekly digital materials containing session content, practice reminders, and audio recordings. The intervention content was fixed during the trial and did not undergo modifications.

IBAP-BP was adapted from a validated mindfulness- and compassion-based program previously applied in diverse populations [17-19]. It targeted key risk factors for PB—perfectionism, limited emotion regulation, and low social support—while fostering self-compassion, emotional regulation, and effective parenting practices [13]. The intervention followed a structured manual ensuring uniform delivery across cohorts. Platform, connectivity, and communication details are provided in Multimedia Appendix 1, and the full module content and practices are summarized in Table S13 in Multimedia Appendix 1.

The AC was a self-guided, home-based program matched in structure and duration. Participants practiced approximately 20 to 30 minutes per day for 8 weeks, combining progressive muscle relaxation with structured journaling and psychoeducational content addressing parental stress, perfectionism, coparenting, adaptive coping, and enjoyment in parenting. Weekly materials and reminders were distributed electronically, and a group chat was available for optional peer support (file formats and delivery logistics are reported in Multimedia Appendix 1).

Adherence in the IBAP-BP group was assessed through session attendance and self-reported home practice, whereas in the AC group, only self-reported practice frequency and duration were collected, given the absence of platform-based tracking.

The WL group received no intervention during the initial 9-month period but was offered IBAP-BP afterward. No restrictions were imposed regarding participation in other interventions during this time.

Data collection and intervention delivery were conducted through secure platforms (Zoom Pro [Zoom Video Communications], WhatsApp [Meta Platforms], and Qualtrics), and participant confidentiality was ensured.

### Instructors

Each group was led by an instructor, supported by an assistant managing the internet-based platform. The team comprised 2 psychologists, 2 psychiatrists, and 1 physician. Four instructors had prior training as mindfulness teachers: 1 in mindfulness-based stress reduction (MBSR), 1 in both MBSR and mindfulness-based cognitive therapy (MBCT), and 2 in MBSR, MBCT, and compassion-based programs. One facilitator did not have formal mindfulness teacher training but had completed an 8-week MBCT program as a participant. This facilitator underwent 6 months of preparation for delivering IBAP, following the Mindfulness-Based Intervention Teacher Assessment Criteria [20]. All instructors completed 8 weekly 2-hour sessions of training on IBAP-BP. All materials, including session guides, content, and audio instructions, were standardized and provided by an external team to ensure consistency across groups.

### Data Collection and Follow-Up

Participants completed internet-based surveys at baseline (preintervention), 3 (end of intervention), 6, and 9 months, with follow-ups extended to 12, 18, and 24 months during an open-label phase after the WL and AC groups were offered. IBAP-BP completion rates were enhanced through email reminders and telephone check-ins conducted by independent assistants. Surveys gathered demographic information (eg, gender, age, educational level, occupation, job role, income range, workplace hierarchy, marital status, and number and gender of children) and assessed primary and secondary outcomes.

The primary outcome was the *Parental Burnout Assessment* (PBA) total score at 9 months, validated in Chile. This 23-item scale measures 4 dimensions: emotional exhaustion, loss of pleasure in the parental role, emotional distancing from children, and self-perception as a parent. Raw scores range from 0 to 138. However, analyses and tables report the average-item metric (range 0‐6), with higher scores indicating greater PB. The use of the average score, rather than the total, avoids the assumption that missing values are equivalent to zero, providing a more accurate representation [21].

Secondary outcomes assessed at 3, 6, or 9 months and during the open phase at 12, 18, and 24 months included PBA, the Five Facets of Mindfulness Questionnaire–15 items [22], and Balance Between Risks and Resources [13]. Self-reported adverse experiences were assessed at 3-, 6-, and 9-month follow-ups using the Unwanted Effects of Meditation Checklist in the IBAP-BP and AC groups only; adverse effects were not assessed in the WL group [23]. Participants reporting significant distress were advised to seek clinical support, and contact information for mental health services was provided. No formal stopping rules were required, given the low-intensity nature of the intervention. In addition, the self-reported number of home practice days and minutes per week, as well as session attendance, were recorded. Other outcomes will be published as secondary data in a separate article (see Multimedia Appendix 1 for details).

### Statistical Analysis

Sample size, power calculation, reliability, and assumption checks are detailed in Multimedia Appendix 1.

Due to scheduling constraints inherent to group-based delivery, a subset of randomized participants withdrew prior to allocation disclosure and before confirming their participation schedule. These individuals were not followed longitudinally and were excluded from the outcome dataset. Accordingly, analyses were conducted using a modified intention-to-treat (mITT) approach, defined a priori as including all randomized participants who did not withdraw before confirming participation and who provided at least 1 postbaseline assessment. Baseline characteristics of included and excluded participants were compared to assess potential bias.

To mitigate potential bias, baseline characteristics of excluded and included participants were compared, and sensitivity analyses were conducted. No interim analyses were prespecified.

The primary end point was PB (PBA total score) at 9 months after the baseline measurement (T3), corresponding to 6 months post intervention. The primary confirmatory analysis consisted of a between-group comparison at 9 months using one-way ANOVA under the mITT framework. To conduct the post hoc analyses, we used the Games-Howell test, rather than the Tukey test, as it is robust to unequal variance and unequal sample size between groups [24]. Effect sizes are reported as mean differences with 95% CI and Cohen *d*.

Missing data in the primary analysis were handled using complete-case data within the mITT sample. Accordingly, the operational primary analysis consisted of a complete-case analysis within the mITT sample, whereas multiple imputation and mixed-effects repeated-measures models were conducted as sensitivity analyses to assess robustness.

Secondary analyses examined between-group differences at 3 (T1) and 6 months (T2), as well as PBA subdimensions, mindfulness, parental risk-resource balance, adverse effects, and adherence-related measures. Sensitivity analyses included (1) a per-protocol analysis including participants in the mindfulness group who attended ≥50% of sessions; (2) an assessment of baseline differences related to follow-up nonresponse; (3) a repeated-measures analysis with imputed data using a generalized mixed model with random coefficients; and (4) a subgroup comparison based on baseline severity, using a cutoff score of ≥84 on the PBA to distinguish between participants classified as treatment (PBA≥84) and prevention (PBA<84) groups.

Exploratory analyses included multilevel structural equation modeling to assess longitudinal intervention effects and cross-lagged panel models to examine potential mediation pathways [25-27]. These analyses were hypothesis-generating and should be interpreted with caution.

Statistical significance was set at *P*<.05. The 9-month PB comparison constituted the prespecified primary confirmatory end point and was therefore not subjected to multiplicity correction. Holm-Bonferroni correction was applied exclusively to secondary and exploratory outcome families to control for type I error [28]. All analyses were conducted in R (R Foundation, v4.3.1). No analysis plan was prospectively preregistered; however, the reported statistics adhere to the recommendations of SAP (Statistical Analysis Plan) guidelines for RCT [29]. Details are provided in Multimedia Appendix 1.

### Ethical Considerations

Ethical approval for this study was granted by the Diego Portales University Ethics Committee on October 27, 2022 (Code 38‐2022). An amendment was subsequently approved on December 20, 2022, to incorporate a third study arm. All participants provided electronic informed consent before enrollment after receiving detailed information regarding the study objectives, procedures, potential risks and benefits, voluntary participation, and the right to withdraw at any time without consequences. Participant privacy and confidentiality were protected throughout the study. Data were collected through secured password-protected platforms, stored in deidentified form, and accessible only to authorized members of the research team. No personally identifiable information was included in the analyses or publication materials. Participants did not receive financial compensation for participation in the study.

## Results

### Baseline Characteristics

A total of 665 participants were enrolled between December 22, 2022, and March 8, 2023. After exclusions and withdrawals, 593 participants were randomized. Of these 593 participants, we found that 39% (n=231) of them presented with high levels of PB (84 points or above), while 61% (n=362) reported low levels of PB (<84 points).

**Figure 1.**
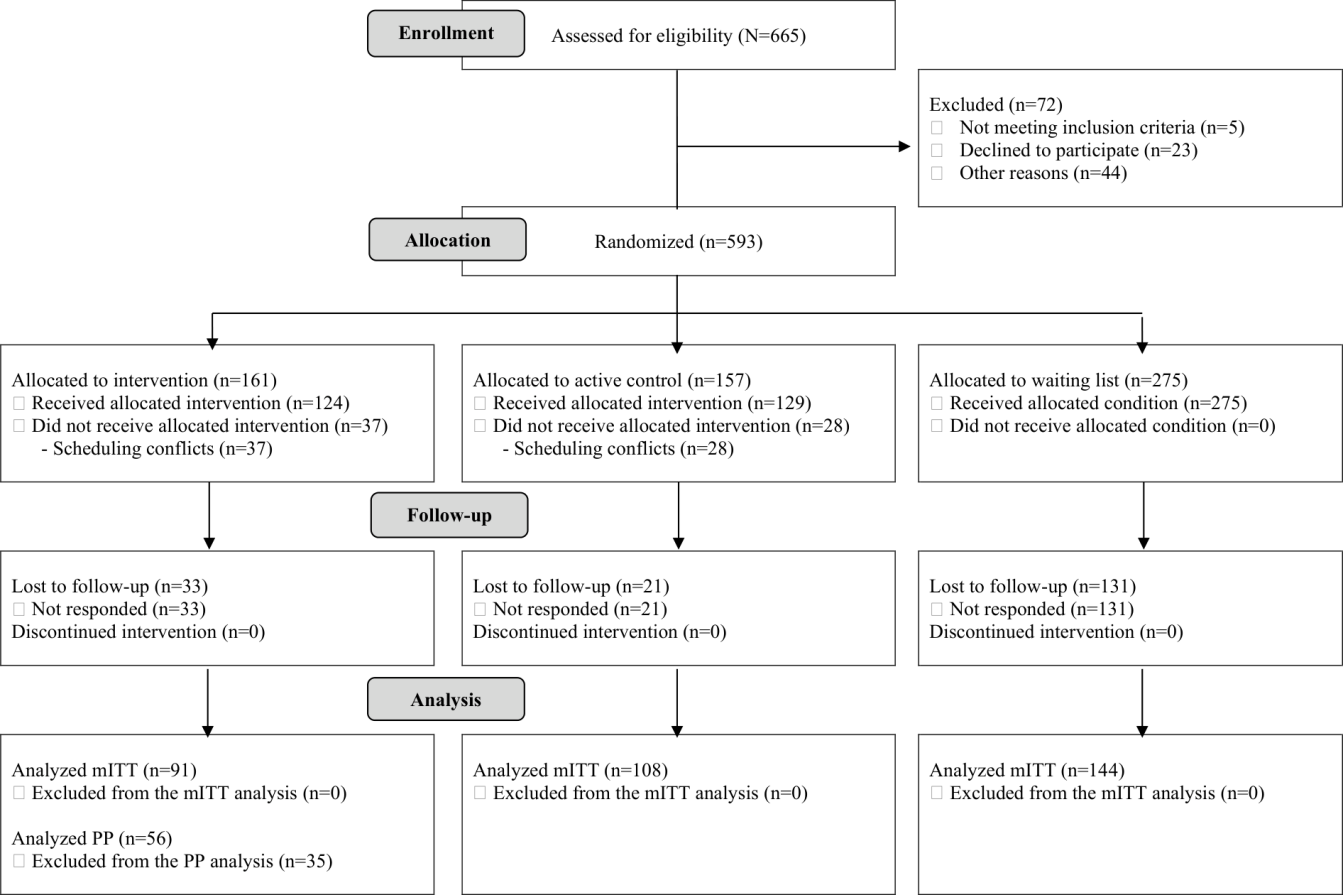
CONSORT (Consolidated Standards of Reporting Trials) flow diagram. mITT: modified intention-to-treat; PP: per-protocol.

Prior to allocation disclosure, 65 participants withdrew due to scheduling conflicts (37 in the IBAP-BP group and 28 in the AC group) and were excluded from the mITT sample. Their slots were dynamically reallocated using participants from the WL group, following the original randomization sequence. The final randomized allocation included 161 participants in the IBAP-BP group (124 initiated the intervention), 157 participants in the AC group (129 initiated the intervention), and 275 participants in the WL group. The mITT analytical sample (n=343) therefore comprised participants who confirmed scheduling after randomization and provided at least 1 postbaseline assessment. A detailed participant flow is provided in the CONSORT diagram (Figure 1).

Participants had a mean age of 38 (SD 5.28) years. Baseline characteristics (age, number of children, workdays, education, and relationship status) were balanced across groups (all *P*>.05 [0.07-0.69]; Table 1), although none followed a normal distribution (all *P*<.001; Table S1 in Multimedia Appendix 1).

**Table 1.** Demographic characteristics of participants in the total sample and by study group: mindfulness- and compassion-based intercare program for parental burnout (IBAP-BP), active control, and waiting list^a^.

Variable	Total sample (N=343)	IBAP-BP (n=91)	Active control (n=108)	Waiting list (n=144)
Age (y), mean (SD)	38.84 (5.28)	38.95 (6.38)	39.18 (4.66)	38.55 (5.01)
Number of children living at home, mean (SD)	1.65 (0.77)	1.73 (0.76)	1.65 (0.78)	1.61 (0.78)
Average age of children (y), mean (SD)	4.96 (4.45)	5.32 (4.85)	4.8 (3.89)	4.86 (4.6)
Number of workdays, mean (SD)	5.01 (0.67)	4.99 (0.69)	5.06 (0.59)	4.99 (0.71)
Number of days working from home, mean (SD)	3.67 (1.46)	3.49 (1.44)	3.53 (1.58)	3.9 (1.36)
Share of child caring responsibilities, mean (SD)	78.72 (16.41)	78.67 (17.42)	79.46 (14.96)	78.19 (16.89)
Educational level, n/N (%)				
Complete school education	7/343 (2%)	2/91 (2%)	1/108 (1%)	4/144 (3%)
Professional education	41/343 (12%)	13/91 (14%)	13/108 (12%)	15/144 (10%)
Bachelor’s degree	164/343 (48%)	32/91 (35%)	50/108 (46%)	82/144 (57%)
Postgraduate degree (eg, MSc)	131/343 (38%)	44/91 (48%)	44/108 (41%)	43/144 (30%)
Relationship status, n/N (%)				
Married	166/343 (48%)	35/91 (38%)	59/108 (55%)	72/144 (50%)
In partnership, but not married	89/343 (26%)	28/91 (31%)	18/108 (17%)	43/144 (30%)
Widow	0/343 (0%)	0/91 (0%)	0/108 (0%)	0/144 (0%)
Divorced	24/343 (7%)	7/91 (8%)	9/108 (8%)	8/144 (6%)
Single	60/343 (17%)	19/91 (21%)	22/108 (20%)	19/144 (13%)
Prefer not to say	2/343 (1%)	2/91 (2%)	0/108 (0%)	2/144 (1%)
Parental burnout, mean (SD)				
Total score	3.38 (1.28)	3.31 (1.39)	3.47 (1.27)	3.37 (1.21)
Parental exhaustion	4.29 (1.39)	4.15 (1.49)	4.36 (1.39)	4.34 (1.33)
Parental identity conflict	2.8 (1.47)	2.84 (1.55)	2.94 (1.49)	2.67 (1.39)
Parental fed up	2.73 (1.38)	2.64 (1.41)	2.77 (1.46)	2.76 (1.31)
Parental emotional distancing	2.91 (1.48)	2.81 (1.59)	3.03 (1.44)	2.89 (1.44)

a Data include age, number of children, child age, workdays, remote workdays, child-caring responsibilities, educational attainment, and relationship status. Values are presented as mean (SD) for continuous variables and percentage for categorical variables. Parental burnout values are presented using the Parental Burnout Assessment (PBA) average-item metric (range 0‐6) rather than the raw total score (0‐138).

### Primary Outcome

**Figure 2.**
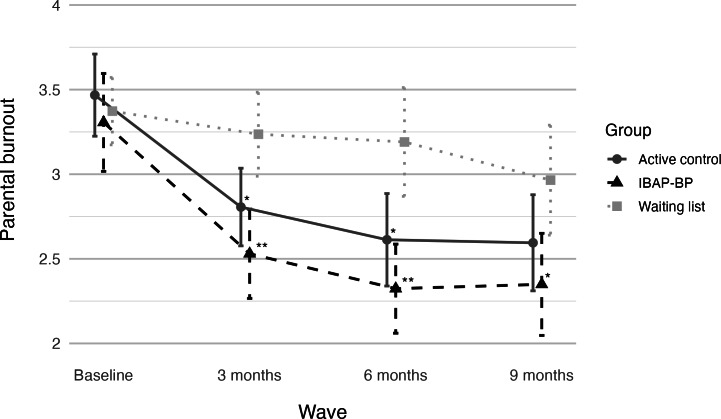
Mean trajectories of parental burnout average-item scores (PBA range 0‐6) per group. Asterisks represent the post hoc Games-Howell test between each intervention group and the waiting list mean level at each wave. Lines represent the CIs. *P<.05, ** *P*<.01. IBAP-BP: mindfulness- and compassion-based intercare program for parental burnout; PBA: Parental Burnout Assessment.

The mITT analysis showed a significant between-group difference in PBA scores at 9 months among participants who completed the 9-month follow-up assessment (n=203; IBAP-BP: n=66, AC: n=73, WL: n=64; ANOVA: *F*_(2, 200)_=4.01, *P*=.02). Post hoc analysis using the Games-Howell test revealed a significant reduction for IBAP-BP versus WL (mean difference 0.62, 95% CI 0.09-1.14), but no significant differences were found between IBAP-BP and AC groups (mean difference −0.25, 95% CI −0.75 to 0.25) or between AC and WL groups (mean difference 0.37, 95% CI −0.14 to 0.88). Reductions from baseline to 9 months were moderate and significant for IBAP-BP (Cohen *d*=0.67, 95% CI 0.40-0.93) and AC (*d*=0.75, 95% CI 0.49-1.01), and they were small and nonsignificant for WL (*d*=0.18, 95% CI −0.07 to 0.41) (Figure 2; Tables S2–S4 in Multimedia Appendix 1).

Attrition analysis showed no baseline PBA differences between completers and dropouts in IBAP-BP and WL (*P*=.96 and *P*=.50 respectively), indicating random losses. In contrast, AC dropouts had lower baseline PBA (*P*=.03), possibly underestimating the effect. No significant differences were observed by age, education, or other baseline sociodemographic variables (*P*>.05 [*P* value ranges between 0.22 and 0.99]).

Multilevel structural equation modeling for the whole mITT sample confirmed the benefits of both interventions over time (N=343; IBAP-BP: n=91; AC: n=108; WL: n=144; IBAP-BP: *b*=−0.20, *P*<.001; AC: *b*=−0.24, *P*<.001). Furthermore, we found a negative main effect of time on PBA (*b*=−0.09, *P*=.01) after controlling for the main effects of both interventions over time, suggesting gradual, but lesser improvement in the WL group (Table S9 in Multimedia Appendix 1). Sensitivity analyses, including per-protocol and multiple imputation for handling missing data, yielded results consistent with the ones already presented (Tables S6-S10 in Multimedia Appendix 1). When examining the sample who complied with the IBAP-BP intervention protocol (>50% of attendance to the sessions), the results show significant differences between the IBAP-BP group and the WL group (mean difference 0.73, 95% CI 0.21-1.26) at 9 months. Furthermore, when using imputed data for handling missing data, we find support for the benefits of the IBAP-BP group (*b*=0.12, *P*=.04), such that there is a significant decrease of PBA over time. We did not find a significant effect of the AC on PBA over time (*b*=−0.11, *P*=.06).

When the sample was divided according to PB levels (231/593, 39% with high PB vs 362/593, 61% without), the intervention showed significant effects only in the group with lower burnout. Specifically, IBAP-BP participants reported greater reductions than those in the WL group (mean difference 0.795, 95% CI 0.275-1.31]), while no significant differences emerged between IBAP-BP and AC (mean difference −0.467, 95% CI −0.939 to 0.004) or between AC and WL (mean difference 0.328, 95% CI −0.261 to 0.916). It is worth noting, however, that reductions from baseline to 9 months were moderate and significant for IBAP-BP in the high-burnout group (Cohen *d*=0.88, 95% CI 0.39-1.35), and these improvements were comparable to those observed in the lower-burnout group (Cohen *d*=0.74, 95% CI 0.40-1.07).

### Secondary Outcome

Significant group differences in PBA scores were detected at 3 months (ANOVA: *F*_2,289_=8.03, *P*<.001) and 6 months (*F*_2,221_=8.57, *P*<.001). Post hoc analysis demonstrated significant improvements in PBA scores for IBAP-BP (third month: mean difference 0.71, 95% CI 0.28-1.14; sixth month: mean difference 0.87, 95% CI 0.37-1.36) and AC (third month: mean difference 0.43, 95% CI 0.03-1.08; sixth month: mean difference 0.58, 95% CI 0.08-1.08) compared to WL at both time points. These findings were consistent in per-protocol analyses (see Table S7 in Multimedia Appendix 1).

Subdimension analyses revealed that IBAP-BP significantly reduced parental exhaustion and emotional distancing at 3, 6, and 9 months compared to WL (third month: mean difference 0.78, 95% CI 0.27-1.29; sixth month: mean difference 1.06, 95% CI 0.49-1.63; ninth month: mean difference 0.74, 95% CI 0.14-1.35). When comparing the IBAP-BP to the WL group, improvements in parental identity conflict and “Fed up” scores were observed at 3 and 6 months (parental identity conflict—third month: mean difference 0.61, 95% CI 0.13-1.08; sixth month: mean difference 0.73, 95% CI 0.18-1.29 and “Fed up”—third month: mean difference 0.64, 95% CI 0.21-1.07; sixth month: mean difference 0.76, 95% CI 0.30-1.22), but these effects were not sustained at 9 months. Only IBAP-BP maintained significant differences across all subdimensions at 9 months in the per-protocol analysis (see Table S7 in Multimedia Appendix 1). Following the Holm-Bonferroni correction applied to secondary outcome families, differences in secondary subdimension analyses at 9 months were no longer statistically significant.

### Safety Analysis and Adherence

Adverse experiences were assessed at the 3-, 6-, and 9-month follow-up waves in the 2 active intervention groups. The intervention demonstrated a favorable safety profile. Self-reported adverse event rates were low and comparable across groups: 0.0%, 1.4%, and 1.5% for IBAP-BP at 3, 6, and 9 months, respectively, compared to 3.6%, 2.5%, and 2.7% for AC. No severe adverse events were reported (*P*=.62, Table 2).

**Table 2.** Self-reported adverse experiences assessed at 3-, 6-, and 9-month follow-up^a^.

Variable	IBAP-BP^b^			Active control		
	3rd month (n=80), n (%)	6th month (n=71), n (%)	9th month (n=66), n (%)	3rd month (n=102), n (%)	6th month (n=79), n (%)	9th month (n=73), n (%)
Reported adverse effects	—^c^	1 (1.4)	1 (1.5)	3 (2.9)	2 (2.5)	2 (2.7)
Greater emotional pain	—	1 (1.4)	1 (1.5)	—	1 (1.3)	—
Increased self-criticism	—	—	1 (1.5)	1 (1)	—	1 (1.4)
Greater fear/anxiety/depression	—	1 (1.4)	1 (1.5)	—	2 (2.5)	2 (2.7)
Lack of direction in life	—	1 (1.4)	1 (1.5)	—	—	1 (1.4)
Lower motivation in life	—	1 (1.4)	—	—	—	—
Feeling of needing something more, of lacking something, etc	—	1 (1.4)	1 (1.5)	1 (1)	1 (1.3)	1 (1.4)
Increased mental confusion	—	1 (1.4)	1 (1.5)	—	1 (1.3)	—
Lack of interest in your surroundings	—	—	—	1 (1)	—	—
Boredom	—	—	—	—	—	—
Need to meditate continuously	—	—	—	—	—	—
Feeling that time not spent meditating is wasted	—	—	—	—	—	—
Restlessness/anxiety when not practicing formal meditation	—	1 (1.4)	—	—	1 (1.3)	—
Increased criticism of others	—	—	—	—	—	—
Greater awareness of one’s negative traits	—	—	1 (1.5)	1 (1)	—	—
Feeling of being superior to others/better than them	—	—	—	—	—	—
Feelings of boredom caused by people	—	—	—	—	—	—
Feelings of lack of interest in others	—	—	—	1 (1)	—	1 (1.4)
Lack of interest in people’s conversations	—	1 (1.4)	—	1 (1)	—	—
Feeling that only people who meditate have value	—	—	—	—	—	—
Feeling of being alienated from society	—	—	—	—	—	—
Hypersensitivity/rejection of urban life	—	—	1 (1.5)	1 (1)	—	—
Difficulty feeling comfortable in the world	—	1 (1.4)	1 (1.5)	1 (1)	—	—
Other (eg, body pain when performing the exercises, guilt for not performing the exercises)	—	1 (1.4)	—	1 (1)	2 (2.5)	—

a The data reflect the number of participants reporting adverse effects across 3 assessment waves in the IBAP-BP and active control groups. Values represent percentages of participants reporting each self-reported adverse experience at each assessment wave. Adverse experiences were assessed only in the IBAP-BP and active control groups at 3-, 6-, and 9-month follow-up.

b IBAP-BP: mindfulness- and compassion-based intercare program for parental burnout.

c Not applicable.

Assistant attendance of the 124 participants enrolled in the IBAP-BP group averaged 3.48 (SD 2.93) sessions, with a range between 0 and 8 sessions. In addition, 48% (n=59) of participants completed more than 50% of the sessions, and 10% (n=13) completed 100% of the program. Self-reported adherence to home practice averaged 2.92 (SD 2.02) days per week and 28.51 (SD 20.38) minutes per week in the IBAP-BP group immediately after the intervention, 1.83 (SD 1.73) days per week and 21.78 (SD 19.81) minutes per week at 6 months, and 1.97 (SD 1.98) days per week and 22.09 (SD 25.69) minutes per week at 9 months. In turn, for the AC, the self-reported practice averaged 2.11 (SD 1.90) days per week and 31.06 (SD 59.49) minutes per week immediately after the intervention, 1.88 (SD 1.97) days per week and 24.53 (SD 28.67) minutes per week at 6 months, and 1.36 (SD 1.53) days per week and 16.75 (SD 17.44) minutes per week at 9 months. In both groups, we observe a steady decline of practice at home over time.

### Exploratory Mediation Analysis

We tested 2 classes of mediators: parental risks or resources and the 5 mindfulness facets. IBAP-BP significantly increased common and specific parental resources (*b*=0.50, *P*=.03 and *b*=0.52, *P*<.01 respectively). In contrast, AC had no effect on either common (*b*=0.20, *P*=.93) or specific resources (*b*=0.33, *P*=.10). However, neither changes in common nor specific resources were associated with changes in PBA scores, suggesting that they did not mediate the intervention effects (Table S11 in Multimedia Appendix 1).

Regarding the 5 facets of mindfulness, IBAP-BP increased the describe (*b*=0.24, *P*=.04) and observe (*b*=0.30, *P*=.008) dimensions and reduced present-moment awareness (*b*=−0.39, *P*<.002). AC increased describe (*b*=0.24, *P*=.03) and reduced awareness (*b*=−0.31, *P*=.01). Nonjudgment postintervention predicted higher PBA scores at 3 months (*b*=0.16, *P*=.02) and observe at 3 months predicted higher PBA at 9 months (*b*=0.34, *P*=.01). The only potential mediation found was an indirect effect of IBAP-BP on PBA reduction at 9 months via postintervention increase in observe—though in the opposite direction of expectations (Table S12 in Multimedia Appendix 1).

## Discussion

### Main Result

This study evaluated an internet-based mindfulness- and compassion-based intervention for PB against AC and WL groups. IBAP-BP showed moderate-to-large improvements versus the WL at 3, 6, and 9 months, but not consistent superiority over the AC. The AC—comprising relaxation, journaling, psychoeducation, and group support—was associated with reductions in burnout up to 3 months after the intervention. However, these effects were not sustained at a longer 9-month follow-up, suggesting a potentially low-cost and scalable, but transient, alternative.

Despite an expected 50% attrition rate, sensitivity analyses confirmed the robustness of the results. Effect sizes increased in per-protocol and imputed analyses, and no systematic attrition patterns were observed. Self-reported adverse events were rare, mild, and comparable across IBAP-BP and AC.

Exploratory mediation analysis showed increased mindfulness among IBAP-BP participants, but effects were inconsistent and inversely related to the nonjudgment and observe facets. No mediation effects were found for parental risks or resources.

### Previous Evidence

Our findings align with prior research demonstrating the effectiveness of mindfulness- and compassion-based interventions in reducing PB. Meta-analyses of related constructs, such as parental stress, have yielded comparable results. Notably, less structured approaches such as those used in our AC group have also shown utility in stress reduction [4,10,14].

This study builds on previous trials by addressing methodological limitations, including the absence of AC and WL, limited follow-up durations (<3 mo), and self-enrollment randomization procedures [10,14,15]. The AC group showed short-term benefits up to 3 months, which diminished over time, contrasting with the sustained effects of IBAP-BP. The inclusion of a WL group clarified the natural progression of PB, revealing modest improvements even without intervention.

To enhance internal validity, we implemented centralized randomization with concealed allocation, reducing the potential for selection bias. In contrast, earlier studies that relied on self-enrollment or site-based randomization may have introduced uncertainty in sequence generation and allocation concealment, which can modestly inflate effect sizes by 7%10% [30,31].

Attrition rates (30%‐50%) were similar to those reported in comparable internet-based interventions [18,19]. A streamlined single-stage recruitment process facilitated accessibility. Among participants who initiated the allocated intervention, retention defined as contributing at least 1 follow-up assessment was 83.7% in the AC group and 73.3% in the IBAP-BP group. These rates exceed those typically observed in multistage recruitment designs and are consistent with prior studies, possibly reflecting proactive follow-up procedures (email and telephone reminders) [10,14,32]. Notably, attrition in the AC group was associated with lower baseline burnout, suggesting potential differential dropout by symptom severity, which may have biased estimates toward greater apparent effectiveness in this group.

The low rate and mild nature of adverse events are consistent with prior literature on MBIs. Transient experiences, such as increased emotional distress, anxiety, or self-criticism, are commonly reported, particularly during the early stages of practice. These effects are generally short-lived and rarely lead to discontinuation or the need for clinical intervention [23].

The study also introduces innovations in delivery and focus. While prior interventions were primarily face-to-face [10,14,15], our internet-based approach improved accessibility while maintaining efficacy. In Chile, where average commute times are lengthy (44-54 min per trip) and inversely correlated with income (*R*²=0.74), internet-based delivery addresses logistical and equity challenges [33].

The AC showed benefits up to 3 months after the intervention, particularly among participants with higher baseline burnout. This suggests that flexible, peer-supported approaches may serve as an initial step within a stepped-care framework. Its components—relaxation, journaling, psychoeducation, and group support—represent established stress-reduction strategies, supporting its role as a therapeutic comparator rather than a neutral control [34-37].

Adherence patterns may have contributed to these differences. IBAP-BP, which combines structured group sessions with home practice, showed more stable engagement, whereas the self-guided AC demonstrated greater variability and decline over time. Prior evidence indicates that strategies such as text reminders and peer coaching can enhance adherence and support sustained engagement [38], which may be particularly relevant for less structured interventions.

The focus on teleworking mothers—100% employed at baseline, compared to 76% to 77% in prior studies—reflects a pragmatic approach aligned with funding priorities. Teleworking mothers may face a higher-risk context for PB. This includes blurred work-family boundaries, increased role conflict, and reduced recovery time—factors consistently linked to emotional exhaustion and parental stress. These conditions may elevate baseline burnout and influence responsiveness to self-regulation–focused interventions [2,16]. While the Latin American socioeconomic context differs from European samples, participants had similar educational levels and marital statuses [10,14]. Although generalizability to lower socioeconomic groups may be limited, effect sizes remained consistent.

IBAP-BP is distinguished by unique components: the “moment of care” priming technique (validated in meta-analyses [39]), compassion exercises for individuals and dyads, habit-formation strategies, and an “inter-care plan” targeting multiple life domains (physical, emotional, social, economic, ethical, and spiritual) [17]. These elements support a comprehensive and sustainable model for reducing PB.

### Mechanisms

Both interventions shared core behavioral ingredients—structured daily practice, psychoeducation, and elements of social support—which are established mechanisms for stress reduction and may account for the comparable outcomes between groups [34-37]. Contrary to standard models of MBIs—which emphasize acceptance, present-moment awareness, and decentering—no consistent mediation effects were found across the 5 mindfulness facets [40-42]. Unexpectedly, increases in nonjudgment and observe were associated with higher PB at follow-up. This may reflect measurement limitations, as certain facets (particularly *observe*) may capture heightened awareness of distress rather than adaptive regulation [43]. Alternatively, unmeasured mechanisms, such as compassion, may account for these associations. The 3-month assessment intervals may have been insufficient to capture the temporal dynamics of mediation, potentially obscuring short-term or delayed effects, as previous research suggests that changes in mindfulness may occur over shorter periods. Statistical suppression or multicollinearity among mindfulness facets may have contributed to directionally inconsistent associations [44,45]. Finally, prior studies have not conclusively demonstrated that mindful parenting mediates changes in PB or in the balance of risks and resources [10], and they do not provide clear support for mindfulness facets as mediators in this context.

### Limitations

Strengths include its design with AC and WL, high retention, culturally tailored content, and real-world recruitment. This is also the first Latin American study on PB, expanding regional representation. Limitations include a lack of adherence monitoring in the AC group and the inability to blind participants. Additionally, loss to follow-up may have reduced confidence in the robustness of the results.

The use of an mITT approach represents a key limitation. Participants who withdrew prior to confirming participation scheduling (n=65) were excluded from analyses, which deviate from strict intention-to-treat principles and may introduce selection bias. In particular, this procedure may limit comparability between randomized groups and reduce generalizability, as individuals with greater logistical constraints or lower availability may be underrepresented in the analytical sample. However, baseline characteristics did not differ significantly between excluded and included participants, and sensitivity analyses yielded consistent results, supporting the robustness of the findings.

Importantly, the third study arm was prospectively approved by the ethics committee prior to participant enrollment through a formal protocol amendment. However, the trial was registered after enrollment had begun, and the third arm was not included in the initial registry entry due to an administrative oversight, being added only in a subsequent update. No primary or secondary outcomes reported in this paper were modified following registration, and all analyses were conducted according to the prespecified protocol. A detailed reconciliation of the original protocol, subsequent amendments, and registration timeline is provided in Multimedia Appendix 1. Nevertheless, this deviation from prospective registration standards may affect perceived transparency and introduce a potential risk of selective reporting bias.

The unexpectedly strong response in the AC group, combined with a sample size powered for larger effects (26%, *d*=0.71), may have limited detection of smaller differences (eg, the observed 10%). Comorbid conditions (eg, depression and posttraumatic stress disorder) or concomitant treatments were not controlled.

Finally, the exclusive inclusion of highly educated, teleworking mothers may limit generalizability. This group may present a distinct burnout profile related to work-family boundary blurring and role overload. Findings may not extend to fathers, unemployed caregivers, or parents of younger children, who may experience different stressors and intervention responses [2,16]. In addition, participation may have been shaped by socioeconomic, digital, and cultural factors, as higher education, income, and digital literacy are associated with greater uptake of digital interventions, while cultural context may influence engagement with mindfulness-based approaches, potentially limiting the inclusion of more vulnerable groups and widening health disparities [46-48].

### Future Research

Future studies should use multicenter designs across diverse settings, test different delivery formats (eg, in-person), and assess cost-effectiveness. Shorter or more flexible programs may improve adherence. Flexible, peer-supported, or stepped-care approaches also appear feasible and warrant further investigation. Including fathers and broader employment contexts would enhance generalizability, while more frequent assessments could help elucidate mechanisms of change.

### Clinical Implications

The internet-based IBAP-BP offers a scalable, accessible, and effective intervention compared with no-treatment conditions, making it especially valuable in resource-limited settings. Its internet-based format addresses barriers such as facilitator scarcity and geographical constraints. From a public health perspective, the program could enhance family well-being and reduce the burden of PB. However, given the absence of clear superiority over an active comparator, flexible, peer-supported, low-cost options—such as the AC program combining relaxation, journaling, psychoeducation, and group support—may represent clinically meaningful alternatives. A stepped-care approach could help bridge the gap toward more intensive and sustainable programs such as IBAP-BP.

## Supplementary material

10.2196/87416 Multimedia Appendix 1 Supplementary materials, including detailed methods; protocol deviations and trial registration reconciliation; recruitment and randomization procedures; intervention content and delivery; statistical and sensitivity analyses; mediation models; supplementary tables and figures; and additional outcome data.

10.2196/87416 Checklist 1 CONSORT checklist.

10.2196/87416 Checklist 2 TIDieR checklist.

## Abbreviations

AC: active control
CONSORT: Consolidated Standards of Reporting Trials
CONSORT-EHEALTH: Consolidated Standards of Reporting Trials of Electronic and Mobile Health Applications and Online Telehealth
IBAP-BP: mindfulness- and compassion-based intercare program for parental burnout
MBCT: mindfulness-based cognitive therapy
MBI: mindfulness-based intervention
MBSR: mindfulness-based stress reduction
mITT: modified intention-to-treat
PB: parental burnout
PBA: Parental Burnout Assessment
RCT: randomized controlled trial
SAP: Statistical Analysis Plan
TIDieR: Template for Intervention Description and Replication
WL: waitlist control
