# Supplementary material for: An Internet-Based Mindfulness- and Compassion-Based Intercare Program for Reducing Parental Burnout: Randomized Controlled Trial
Source: J Med Internet Res. 2026 Jun 25;28:e87416. doi: 10.2196/87416 (PMC13299005; doi:10.2196/87416)
Supplement: Multimedia Appendix 1 [file jmir-v28-e87416-s001.docx]

# Supplementary appendix

## Methods

Some deviations from the original protocol are clarified here. The initial sample size and power calculation estimated a total of 212 participants (106 per group). However, considering that each intervention group could accommodate 25 participants and we had five trained instructors, we decided to extend the target sample to 125 participants per arm.

Prior to enrollment, we decided to include a third arm—a waitlist control group—as part of an observational comparison. An amendment was submitted and approved by the Ethics Committee to reflect this change.

Although the estimated representative sample size for teleworking mothers in Chile was 768, we ultimately recruited 665 participants, of whom 72 were excluded. While this was sufficient for comparative analyses across the three study arms, it was not enough to serve as a representative sample of teleworking mothers in Chile for the purposes of an observational cohort study.

### Trial Registration Acknowledgement

We acknowledge discrepancies in the timing and completeness of trial registration. The original study protocol received ethical approval on 27 October 2022, followed by an amendment on 20 December 2022 incorporating a third arm (waitlist control). A subsequent amendment including the microbiota substudy was approved on 31 January 2023.

Participant enrollment occurred between 22 December 2022 and 8 March 2023. The intervention was delivered in two waves between 7–14 April 2023 and concluded on 6 June 2023. Trial registration was submitted on 15 April 2023, during the first week of intervention delivery.

Due to an administrative oversight, the third arm was not included in the initial registration and was added on 15 August 2023. We acknowledge that this does not meet prospective registration standards. However, all study arms and outcomes were defined in the protocol prior to enrollment, and no post hoc changes to outcomes or analyses were made. Randomization procedures were implemented as planned before intervention initiation.

This discrepancy may introduce a risk of perceived selective reporting bias, which is acknowledged and discussed in the main manuscript.

A reconciliation of the originally approved design, subsequent amendments, timing of changes, and protocol deviations is provided in Supplementary Table 14.

### Participants

Eligibility criteria included women aged ≥18 years, teleworking at least one day per week, and residing with at least one child. These criteria were pragmatically chosen, reflecting evidence that teleworking increases the risk of burnout and aligning with the funding institution's requirements.2,20 This approach reflects a common format used in community-based programs. No protocol restrictions were applied concerning other treatments. Exclusion criteria included self-reported severe depression, substance abuse, or psychotic disorder.

Participants were recruited between December 22, 2022, and March 8, 2023, through a combination of paid and institutional outreach strategies. A targeted advertising campaign was launched on Instagram using Meta’s advertising tools, selecting users in Chile who self-identified as women and expressed interests related to mindfulness, meditation, well-being, mental health, maternity, and parenting. In parallel, recruitment was conducted through partnerships with five companies affiliated with the Chilean Occupational Safety Institute (IST), which distributed the invitation via internal mailing lists. Additional dissemination was carried out through the Chilean Medical Association's social media channels and by physicians with strong social media presence in the area of maternal mental health. All advertisements invited individuals to participate in a study evaluating the effectiveness of an intervention to reduce parental burnout. Interested participants were directed to an internet-based platform where they completed a contact form and provided electronic informed consent. Those who consented were then sent a baseline questionnaire to determine eligibility and proceed with enrollment.

### Randomization and Blinding

Participants were centrally randomized by an independent researcher using a computer-generated sequence, with allocation concealment maintained until assignment. Stratification was performed based on baseline parental burnout severity (PBA ≥84), and no blocking was applied.

Due to logistical constraints related to group-based delivery, the IBAP-BP and active control (AC) arms had fixed capacity (target n=125 each), determined by the maximum number of sessions that could be delivered in parallel. The waitlist (WL) group had no capacity restriction. Randomization was implemented across the full eligible sample, with allocation to intervention arms occurring until capacity was reached and remaining participants assigned to the WL group. Centralized assignment allowed all participants, including those initially allocated to WL, to remain in the randomization pool.

Prior to allocation disclosure, participants were required to confirm their availability by selecting one of the predefined session schedules, without knowledge of their assigned group. Participants who reported scheduling conflicts and withdrew before allocation disclosure were excluded prior to intervention initiation. Vacant intervention slots were subsequently filled using participants from the WL pool, following the original randomization sequence. This replacement process was applied iteratively until all intervention slots were filled.

As a result, the total number of enrolled participants exceeded the initially planned sample size, and the final allocation ratio was slightly unbalanced across groups. Baseline parental burnout levels were compared across the three groups using ANOVA to confirm equivalence prior to intervention.

Allocation remained concealed until the point of assignment. Following assignment, ten independent research assistants contacted participants via email and telephone to coordinate participation. For the IBAP-BP group, this involved scheduling two-hour live group sessions; for the AC group, participants were assigned to concurrent self-guided home-based activities. These procedures ensured allocation concealment prior to assignment and minimized the risk of selection or scheduling bias.

Due to the nature of the intervention, blinding of participants and instructors was not feasible. However, data analysts and one investigator remained blinded throughout the study. After completion of the primary nine-month outcome assessment, participants in the AC and WL groups were offered access to the IBAP-BP intervention in an open-label phase (Supplementary Figure 1).

**Procedure**

The IBAP-BP intervention consisted of eight weekly two-hour group sessions delivered internet-based via Zoom Pro, led by trained instructors. In addition, participants were asked to complete 20–30 minutes of daily home practice, including formal meditations and structured journaling. The intervention was conducted in Chile, and all participants had access to a stable internet connection, a microphone, and an electronic device to attend sessions and participate in the WhatsApp support group. The program was standardized across cohorts, with no individual tailoring, although participants were encouraged to follow their own rhythm when engaging in the exercises. No modifications were made during implementation.

Each week, participants received a digital document via email containing the session’s content, reminders for the home practices, and guided meditation audios in MP3 format. Five cohorts of 25 participants each completed the program. IBAP-BP was adapted from a validated intervention previously applied to diverse populations, grounded in established theoretical frameworks.17,18,21 It targets key risk factors for parental burnout—such as perfectionism, limited emotional regulation, and lack of social support—and fosters self-compassion, emotional regulation, and effective parenting practices.19

The eight modules covered the following topics: (1) Mindfulness and autopilot, (2) Observing the mind and self-care, (3) Contact with experience, (4) Shared humanity and working with discomfort, (5) Acceptance and gratitude, (6) Self-compassion and compassionate communication, (7) Mutual care, and (8) Integration and planning. Formal meditation practices included mindful eating, body scan, breath awareness, mindful stretching, walking meditation, sound and thought observation, open awareness, compassion, and gratitude practices. Brief exercises such as the "self-care moment" and "breathing space" meditations were also included. Additional themes addressed parenting values, time management, emotional regulation, and mindful parenting strategies.

A structured intervention manual provided step-by-step instructions for each session, exercise, and meditation, and scripted guidance was used to ensure consistency. All instructors received prior training, and external assistants were responsible for material distribution. Although fidelity was not assessed through direct observation, all participants received the same meditation audio guides. Materials are available at reasonable request. Further details are provided in Supplementary Table 13.

The active control group received a self-guided, home-based program designed to match the structure and duration of the IBAP-BP intervention. Participants were instructed to engage in approximately 20–30 minutes of daily practice over eight weeks, including two core components: (1) Jacobson’s progressive muscle relaxation (guided audio recordings of 10 or 20 minutes), and (2) structured journaling based on psychoeducational topics such as parental stress, perfectionism, co-parenting, adaptive coping strategies, and enjoyment in parenting. Weekly materials were delivered via email by trained research assistants and included PDFs, audio files, and instructions. Weekly reminders were sent via email and WhatsApp, and a WhatsApp group was created for optional peer support. The program was fully remote, required participants to have a device with internet access, and was standardized without personalization.

Adherence in the IBAP-BP group was assessed through session attendance and self-reported home practice, whereas in the active control group only self-reported practice frequency and duration were collected, given the absence of platform-based tracking.

The waitlist control group did not receive any intervention during the initial nine-month study period but was offered participation in the IBAP-BP program afterward. No restrictions were applied regarding participation in other interventions during the waitlist period.

### Primary outcomes

The primary Outcome was Parental Burnout Assessment (PBA) total score at nine months validated in Chile. This 23-item scale measures four dimensions: emotional exhaustion, loss of pleasure in the parental role, emotional distancing from children, and self-perception as a parent. Raw scores range from 0 to 138; however, analyses and tables report the average-item metric (range 0–6), with higher scores indicating greater parental burnout. The use of the average score, rather than the total, avoids the assumption that missing values are equivalent to zero, providing a more accurate representation ^1^.

### Secondary outcomes

Secondary outcomes assessed at three, six or nine month, and during the open phase at 12, 18 and 24 months included PBA, Five Facets of Mindfulness Questionnaire - 15 items (FFMQ-15) ^2^, Balance Between Risks and Resources (BR2) ^3^. Adverse effects were assessed only in the intervention groups using the Unwanted Effects of Meditation Checklist (UEM) and reflect self-reported experiences without formal attribution to the intervention. and adverse effects, which were reported through the ^4^. Participants reporting significant distress were advised to seek clinical support, and contact information for mental health services was provided. No formal stopping rules were required given the low-intensity nature of the intervention. In addition, self-reported number of home practice days and minutes per week, as well as session attendance, were measured.

Other secondary outcomes will be reported in a separate article. These outcomes were selected to address secondary research questions. Specifically, we aimed to compare the effect of the intervention on work-related burnout and examine differences with parental burnout. To this end, work engagement was included as a work-specific outcome, while quality of life was assessed as a broader comparator. The following instruments were used: European Quality of Life-5 Dimensions (EQ-5D)^5^, Utrecht Work Engagement Scale - 9 items (UWES-9)^6^, Maslach Burnout Inventory (MBI) - Emotional Exhaustion Scale^5^.

Additional measures were included to explore potential mediators, based on theory-driven constructs such as decentering, emotional regulation, mindfulness, and personality traits, as well as psychosocial factors related to work and family contexts. These included: Decentering scale^7^, Emotion Regulation Questionnaire (ERQ)^8^, Psychosocial Factors Questionnaire – Work-Family Balance Extract (PSF-Q75)^9^, and Big five inventory Short Questionnaire Extraversion and Neuroticism Extract^10^.

Finally, as an exploratory secondary research question, we examined the relationship between the intervention and gut microbiota. We hypothesized that the mindfulness-based intervention, through a top-down mechanism targeting stress regulation, would lead to changes in microbiota composition—specifically, increased diversity and the enrichment of subpopulations associated with psychological well-being. To investigate this, a subsample of 16 participants from each intervention group (total N = 32) was selected for additional secondary analyses, including microbiota profiling, respiratory sinus arrhythmia as an indirect marker of vagal tone, and focus groups to assess qualitative outcomes.

### Statistics

Sample size was calculated for independent groups with continuous variables, assuming a 1:1 recruitment ratio, alpha = 0.05, power = 95%, and a 50% dropout rate. Based on prior intervention studies (M=86.49, SD=29.77) and an expected reduction of 26%, a total of 212 participants were required (106 per group).

Additionally, we decided to introduce a third arm (waiting list) to examine the natural course of parental burnout, enhance the study’s robustness, and address methodological gaps identified in previous research. This modification was approved by the ethics committee, and all three arms were included in the randomization process. For the additional observational cohort (waiting list arm), the sample size was calculated to be representative of Chilean teleworking mothers. Considering a population of approximately 260,000 mothers working remotely, a 95% confidence level, a 5% margin of error, and a 50% dropout rate, the required sample size for this group was 768 participants. Although this number exceeds the requirements for a randomized trial, it was chosen to enhance the representativeness of this arm.

To test the hypotheses, several steps were taken. First, the reliability of the scales for each wave was examined using Cronbach’s Alpha. Then, we tested the normal distribution assumption of all variables using the Shapiro-Wilk test. To examine the homoscedasticity assumption between groups, we used Leven’s Test, as it is robust to deviations of normality.

The primary confirmatory analysis consisted of between-group comparisons of parental burnout (PBA total score) at nine months using one-way ANOVA under the modified intention-to-treat framework. Post hoc pairwise comparisons were conducted using the Games–Howell test, selected over Tukey’s test due to its robustness to unequal variances and sample sizes.^11^ Secondary analyses examined group differences at earlier timepoints (3 and 6 months) and across PBA subdimensions.

To assess longitudinal intervention effects, the SEM framework was chosen because it is robust to missing data, especially when Full Information Maximum Likelihood is used in the estimation process.^12^ Here, we first modelled the direct effect of time at the within level and direct effect of the intervention at the between level. Then, the interaction of time and group membership was included. Since group membership is categorical, we created three dummy variables depicting group membership. The waiting list group was used as the baseline; thus, we only included the dummy variables of the mindfulness group and the active control group in the analysis. To assess model fit, we used a combination of well accepted fit indices. Specifically, we used χ^2^ test for absolute fit testing, and the CFI, TLI, RMSEA, and SRMR for local fit testing. A non-significant χ^2^ test, a CFI and TLI larger than 0.95, and an RMSEA and SRMR lower than 0.06 are typically considered indicative of excellent fit.^13^ Here we would expect a significant effect of the interaction between time and having received the mindfulness treatment but no evidence for having participated in the active control group.

To assess longitudinal intervention effects, we used a cross-lagged panel model^14,15^ Here we estimated one model for each class of mediator. We included all autoregressive paths, but only included the direct effect of the intervention. Namely, we only included the path of group membership dummy variables on the dependent variable immediately after the intervention. This is because as a time-invariant predictor, the effect of the interventions in later waves should only exist via their effects on the key variables immediately after the intervention.^16^

Sensitivity analyses were conducted to assess robustness of the primary findings. First, we repeated the key analysis using a subsample of the mindfulness group that completed the intervention protocol (i.e., attended to 50% or more of the group sessions). Second, we whether non-response to follow-up surveys was due to systematic difference in the baseline levels of parental burnout. Specifically, for each follow-up wave, we compared baseline levels of parental burnout between those who completed the follow-up and those who did not. Here, we used Bartlett’s test to assess the homoskedasticity of the variance assumption between both groups and either a T-Test or a Welch’s Test for independent samples to compare the mean levels depending on whether the equal variance assumption held. Finally, we re-estimated the repeated measure analysis imputing missing data and using a generalized mixed model with random coefficients to analyze the new samples. Here, we imputed 10 samples, run a separate model with each imputed sample, and then aggregated the results. Fourth, we conducted a subgroup analysis by baseline parental burnout severity, using a cut-off score of PBA ≥84 to distinguish participants with higher versus lower baseline burnout.

Statistical significance was set at p < 0.05. The 9-month parental burnout comparison constituted the prespecified primary confirmatory endpoint and was therefore not subjected to multiplicity correction. Holm–Bonferroni correction was applied exclusively to secondary and exploratory outcome families to control for type I error. All analyses were conducted in R (v4.3.1). No analysis plan was prospectively preregistered but reported statistic adhere to recommendation of SAP Guidelines for RCT.

**Supplementary Figure 1. Study design**

Study design, illustrating the three study arms, assessment time points, and subsamples for microbiota analysis. The primary outcome was assessed at 9 months, followed by an open-label phase with follow-up evaluations at 12, 18, and 24 months. IBAP-BP = Mindfulness- and compassion-based inter-care program for Parental Burnout.

**
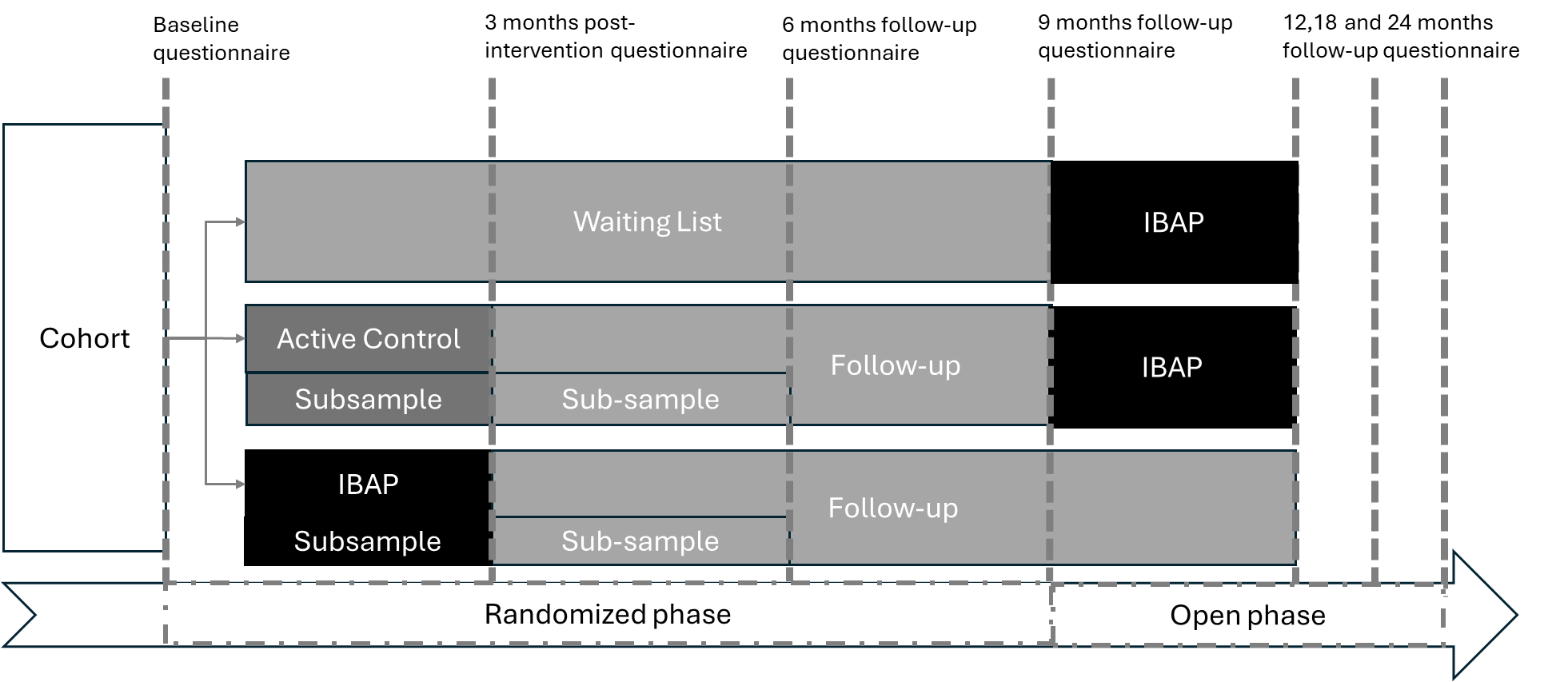
**

| **Supplementary Table 1.**  **Shapiro-Wilk Test for variables per waves**  IBAP-BP = Mindfulness and Compassion-Based Inter-Care Program for Parental Burnout. ** p < 0.01 | | | | |
| --- | --- | --- | --- | --- |
| **Variables** | **Baseline** | **3rd month** | **6th month** | **9th month** |
| *Parental Burnout* |  |  |  |  |
| Full Sample | 0.97** | 0.94** | 0.92** | 0.92** |
| IBAP-BP | 0.95** | 0.89** | 0.89** | 0.87** |
| Active Control | 0.97** | 0.93** | 0.91** | 0.91** |
| Waiting List | 0.97** | 0.96** | 0.95** | 0.95** |
| *Parental Exhaustion* |  |  |  |  |
| Full Sample | 0.98** | 0.97** | 0.96** | 0.95** |
| IBAP-BP | 0.96** | 0.94** | 0.94** | 0.92** |
| Active Control | 0.98** | 0.97** | 0.95** | 0.94** |
| Waiting List | 0.98** | 0.97** | 0.95** | 0.98** |
| *Parental Identity Conflict* |  |  |  |  |
| Full Sample | 0.91** | 0.86** | 0.83** | 0.84** |
| IBAP-BP | 0.91** | 0.82** | 0.79** | 0.76** |
| Active Control | 0.90** | 0.84** | 0.8** | 0.85** |
| Waiting List | 0.91** | 0.89** | 0.88** | 0.89** |
| *Parental Fed Up* |  |  |  |  |
| Full Sample | 0.92** | 0.85** | 0.83** | 0.86** |
| IBAP-BP | 0.9** | 0.77** | 0.82** | 0.79** |
| Active Control | 0.91** | 0.82** | 0.79** | 0.86** |
| Waiting List | 0.93** | 0.90** | 0.89** | 0.91** |
| *Parental Emotional Distancing* |  |  |  |  |
| Full Sample | 0.92** | 0.9** | 0.85** | 0.85** |
| IBAP-BP | 0.90** | 0.86** | 0.82** | 0.79** |
| Active Control | 0.92** | 0.88** | 0.85** | 0.85** |
| Waiting List | 0.92** | 0.93** | 0.89** | 0.88** |

| **Supplementary Table 2.**  Mean (SD), median, and range (min-max) for parental burnout, exhaustion, identity conflict, feeling fed up, and emotional distancing at baseline, 3, 6, and 9 months across the full sample and by intervention group: Mindfulness and compassion-based inter-care program (IBAP-BP), Active Control, and Waiting List. N represents the number of participants at each time point. Parental burnout values are presented using the PBA average-item metric (range 0–6) rather than the raw total score. | | | | | | | | | | | | |
| --- | --- | --- | --- | --- | --- | --- | --- | --- | --- | --- | --- | --- |
|  | **Baseline** | | | **3rd month** | | | **6th month** | | | **9th month** | | |
| **Variable** | **N** | **Mean (SD)** | **Median**  **(Min - Max)** | **N** | **Mean (SD)** | **Median**  **(Min - Max)** | **N** | **Mean (SD)** | **Median**  **(Min - Max)** | **N** | **Mean (SD)** | **Median**  **(Min - Max)** |
| *Parental Burnout* |  |  |  |  |  |  |  |  |  |  |  |  |
| Full Sample | 343 | 3.38 (1.28) | 3.3 (1.13 - 6.65) | 292 | 2.89 (1.26) | 2.65 (1 - 7) | 224 | 2.71 (1.29) | 2.35 (1 - 6.65) | 203 | 2.63 (1.27) | 2.35 (1 - 6.17) |
| IBAP-BP | 91 | 3.31 (1.39) | 3.26 (1.22 - 6.65) | 80 | 2.53 (1.19) | 2.17 (1 - 6.78) | 71 | 2.32 (1.11) | 2.13 (1 - 6.35) | 66 | 2.35 (1.23) | 1.91 (1 - 6.17) |
| Active Control | 108 | 3.47 (1.27) | 3.35 (1.13 - 6.52) | 102 | 2.81 (1.17) | 2.78 (1.13 - 7) | 79 | 2.61 (1.22) | 2.35 (1 - 6.3) | 73 | 2.59 (1.22) | 2.26 (1 - 5.65) |
| Waiting List | 144 | 3.37 (1.21) | 3.26 (1.14 - 6.65) | 110 | 3.24 (1.31) | 3 (1 - 6.57) | 74 | 3.19 (1.39) | 2.73 (1 - 6.65) | 64 | 2.96 (1.3) | 2.72 (1.04 - 5.83) |
| *Parental Exhaustion* |  |  |  |  |  |  |  |  |  |  |  |  |
| Full Sample | 343 | 4.29 (1.39) | 4.33 (1.11 - 7) | 292 | 3.64 (1.46) | 3.50 (1 - 7) | 224 | 3.37 (1.5) | 3.06 (1 - 7) | 203 | 3.22 (1.5) | 3 (1 - 6.89) |
| IBAP-BP | 91 | 4.15 (1.49) | 4.22 (1.22 - 6.78) | 80 | 3.24 (1.49) | 2.94 (1 - 7) | 71 | 2.86 (1.35) | 2.78 (1 - 6.78) | 66 | 2.89 (1.48) | 2.5 (1 - 6.56) |
| Active Control | 108 | 4.36 (1.39) | 4.33 (1.33 - 7) | 102 | 3.52 (1.35) | 3.56 (1.22 - 7) | 79 | 3.31 (1.43) | 3 (1 - 6.44) | 73 | 3.16 (1.49) | 2.78 (1 - 6.78) |
| Waiting List | 144 | 4.34 (1.33) | 4.33 (1.11 - 7) | 110 | 4.02 (1.44) | 3.94 (1 - 7) | 74 | 3.92 (1.55) | 3.56 (1 - 7) | 64 | 3.64 (1.44) | 3.61 (1 - 6.89) |
| *Parental Identity Conflict* |  |  |  |  |  |  |  |  |  |  |  |  |
| Full Sample | 343 | 2.80 (1.47) | 2.33 (1 - 6.83) | 292 | 2.46 (1.4) | 2 (1 - 7) | 224 | 2.33 (1.42) | 1.83 (1 - 6.5) | 203 | 2.25 (1.32) | 1.83 (1 - 6.67) |
| IBAP-BP | 91 | 2.84 (1.55) | 2.5 (1 - 6.67) | 80 | 2.14 (1.27) | 1.67 (1 - 6.83) | 71 | 2.04 (1.22) | 1.67 (1 - 6.5) | 66 | 1.98 (1.22) | 1.67 (1 - 6.33) |
| Active Control | 108 | 2.94 (1.49) | 2.33 (1 - 6.5) | 102 | 2.4 (1.35) | 2.17 (1 - 7) | 79 | 2.18 (1.35) | 1.67 (1 - 6.17) | 73 | 2.25 (1.3) | 2 (1 - 6.67) |
| Waiting List | 144 | 2.67 (1.39) | 2.33 (1 - 6.83) | 110 | 2.75 (1.48) | 2.17 (1 - 6.83) | 74 | 2.77 (1.58) | 2.17 (1 - 6.33) | 64 | 2.52 (1.42) | 2.33 (1 - 6.67) |
| *Parental Fed Up* |  |  |  |  |  |  |  |  |  |  |  |  |
| Full Sample | 343 | 2.73 (1.38) | 2.4 (1 - 7) | 292 | 2.28 (1.3) | 1.9 (1 - 7) | 224 | 2.17 (1.26) | 1.8 (1 - 6.4) | 202 | 2.18 (1.21) | 1.8 (1 - 6.2) |
| IBAP-BP | 91 | 2.64 (1.41) | 2.2 (1 - 6.4) | 80 | 1.94 (1.14) | 1.5 (1 - 6.8) | 71 | 1.82 (.95) | 1.5 (1 - 5.6) | 66 | 1.97 (1.15) | 1.6 (1 - 5.8) |
| Active Control | 108 | 2.77 (1.46) | 2.4 (1 - 6.8) | 102 | 2.23 (1.31) | 1.8 (1 - 7) | 79 | 2.11 (1.3) | 1.6 (1 - 6.4) | 72 | 2.15 (1.16) | 2 (1 - 6.2) |
| Waiting List | 144 | 2.76 (1.31) | 2.6 (1 - 7) | 110 | 2.58 (1.36) | 2.2 (1 - 6.8) | 74 | 2.58 (1.38) | 2.2 (1 - 6.4) | 64 | 2.44 (1.31) | 2.1 (1 - 5.8) |
| *Parental Emotional Distancing* |  |  |  |  |  |  |  |  |  |  |  |  |
| Full Sample | 343 | 2.91 (1.48) | 2.67 (1 - 7) | 292 | 2.54 (1.38) | 2.17 (1 - 7) | 223 | 2.4 (1.39) | 2 (1 - 6.67) | 203 | 2.37 (1.38) | 2 (1 - 6.33) |
| IBAP-BP | 91 | 2.81 (1.59) | 2.33 (1 - 7) | 80 | 2.15 (1.12) | 1.67 (1 - 6.33) | 70 | 2.11 (1.22) | 1.83 (1 - 6.33) | 66 | 2.09 (1.31) | 1.67 (1 - 5.67) |
| Active Control | 108 | 3.03 (1.44) | 2.67 (1 - 6.67) | 102 | 2.41 (1.31) | 2 (1 - 7) | 79 | 2.23 (1.19) | 2 (1 - 6.67) | 73 | 2.32 (1.2) | 2 (1 - 6) |
| Waiting List | 144 | 2.89 (1.44) | 2.5 (1 - 6.67) | 110 | 2.95 (1.53) | 3 (1 - 6.33) | 74 | 2.85 (1.63) | 2.5 (1 - 6.67) | 64 | 2.72 (1.58) | 2.17 (1 - 6.33) |

| **Table 3**  **ANOVA Results Across Waves – Modified Intention to Treat Analysis.**  Results of ANOVA tests for parental burnout and related constructs at different time points: 0 = Baseline, 1 = 3rd month, 2 = 6th month, 3 = 9th month. Levene’s test assesses homogeneity of variance, and ANOVA tests group differences. ** p < 0.01, * p < 0.05; In case Leven’s Test for variance homogeneity between groups was significant, White’s adjustment was used for heteroskedasticity-robust robust standard errors. | | | |
| --- | --- | --- | --- |
| **Variable** | **Wave** | **Levene's Test *F*(*df1, df2*)** | **ANOVA *F*(*df1, df2*)** |
| Parental Burnout | 0 | 2.64 (2, 340), p = 0.07 | .40 (2, 340), p = 0.67 |
| Parental Burnout | 1 | 2.68 (2, 289), p = 0.07 | 8.03 (2, 289), p = 0.00** |
| Parental Burnout | 2 | 3.38 (2, 221), p = 0.04* | 8.57 (2, 221), p = 0.00** |
| Parental Burnout | 3 | .38 (2, 200), p = 0.68 | 4.01 (2, 200), p = 0.02* |
| Parental Exhaustion | 0 | 1.38 (2, 340), p = 0.25 | .69 (2, 340), p = 0.50 |
| Parental Exhaustion | 1 | 1.01 (2, 289), p = 0.37 | 7.42 (2, 289), p = 0.00** |
| Parental Exhaustion | 2 | 1.5 (2, 221), p = 0.22 | 9.77 (2, 221), p = 0.00** |
| Parental Exhaustion | 3 | .04 (2, 200), p = 0.96 | 4.26 (2, 200), p = 0.02* |
| Parental Identity Conflict | 0 | 1.45 (2, 340), p = 0.24 | 1.08 (2, 340), p = 0.34 |
| Parental Identity Conflict | 1 | 1.41 (2, 289), p = 0.25 | 4.63 (2, 289), p = 0.01* |
| Parental Identity Conflict | 2 | 3.09 (2, 221), p = 0.05 | 5.09 (2, 221), p = 0.00** |
| Parental Identity Conflict | 3 | 1.98 (2, 200), p = 0.14 | 2.77 (2, 200), p = 0.07 |
| Parental Fed Up | 0 | .97 (2, 340), p = 0.38 | .27 (2, 340), p = 0.77 |
| Parental Fed Up | 1 | 2.38 (2, 289), p = 0.09 | 5.88 (2, 289), p = 0.00** |
| Parental Fed Up | 2 | 2.95 (2, 221), p = 0.05 | 7.12 (2, 221), p = 0.00** |
| Parental Fed Up | 3 | 2.03 (2, 199), p = 0.13 | 2.53 (2, 199), p = 0.08 |
| Parental Emotional Distancing | 0 | .83 (2, 340), p = 0.44 | .57 (2, 340), p = 0.57 |
| Parental Emotional Distancing | 1 | 7.81 (2, 289), p = 0.00** | 8.65 (2, 289), p = 0.00** |
| Parental Emotional Distancing | 2 | 6.93 (2, 220), p = 0.00** | 5.18 (2, 220), p = 0.00** |
| Parental Emotional Distancing | 3 | 2.43 (2, 200), p = 0.09 | 3.51 (2, 200), p = 0.03* |

| **Table 4.**  **Post-Hoc ANOVA Analysis to Examine Between Group Differences – Modified Intention to Treat**  Pairwise comparisons of parental burnout and related constructs across intervention groups: Mindfulness and Compassion-Based Inter-Care Program for Parental Burnout (IBAP-BP), Active Control, and Waiting List at baseline, 3, 6, and 9 months. Parental burnout values are presented using the PBA average-item metric (range 0–6) rather than the raw total score. Values represent mean differences with 95% confidence intervals (CIL, CIU). Positive values indicate lower scores in the first group compared to the second. ** p < 0.01, * p < 0.05. Games-Howell test was used as a post hoc analysis as it is robust to unequal variance and unbalanced sample size between groups. | | | | | | |
| --- | --- | --- | --- | --- | --- | --- |
| Variable | Group 1 | Group 2 | Baseline (CI_L_, CI_U_) | 3rd month (CI_L_, CI_U_) | 6th month (CI_L_, CI_U_) | 9th month (CI_L_, CI_U_) |
| Parental Burnout | IBAP-BP | Waiting List | 0.07 (-0.35, 0.49) | 0.71 (0.28, 1.14)** | 0.87 (0.37, 1.36)** | 0.62 (0.09, 1.14)* |
| Parental Burnout | Active Control | Waiting List | -0.09 (-0.47, 0.28) | 0.43 (0.03, 0.83)* | 0.58 (0.08, 1.08)* | 0.37 (-0.14, 0.88) |
| Parental Burnout | Active Control | IBAP-BP | -0.16 (-0.61, 0.29) | -0.28 (-0.69, 0.14) | -0.29 (-0.74, 0.16) | -0.25 (-0.74, 0.25) |
| Parental Exhaustion | IBAP-BP | Waiting List | 0.19 (-0.26, 0.65) | 0.78 (0.27, 1.29)** | 1.06 (0.49, 1.63)** | 0.74 (0.14, 1.35)* |
| Parental Exhaustion | Active Control | Waiting List | -0.01 (-0.42, 0.40) | 0.50 (0.05, 0.95)* | 0.61 (0.04, 1.18)* | 0.47 (-0.12, 1.07) |
| Parental Exhaustion | Active Control | IBAP-BP | -0.21 (-0.69, 0.28) | -0.28 (-0.79, 0.22) | -0.45 (-0.99, 0.09) | -0.27 (-0.87, 0.33) |
| Parental Identity Conflict | IBAP-BP | Waiting List | -0.17 (-0.64, 0.3) | 0.61 (0.13, 1.08)** | 0.73 (0.18, 1.29)** | 0.54 (-0.01, 10.09) |
| Parental Identity Conflict | Active Control | Waiting List | -0.27 (-0.70, 0.17) | 0.35 (-0.11, 0.81) | 0.59 (0.02, 1.15)* | 0.27 (-0.29, 0.82) |
| Parental Identity Conflict | Active Control | IBAP-BP | -0.10 (-0.61, 0.41) | -0.25 (-0.72, 0.21) | -0.14 (-0.64, 0.35) | -0.27 (-0.78, 0.23) |
| Parental Fed Up | IBAP-BP | Waiting List | 0.12 (-0.31, 0.55) | 0.64 (0.21, 1.07)** | 0.76 (0.30, 1.22)** | 0.47 (-0.04, 0.98) |
| Parental Fed Up | Active Control | Waiting List | -0.01 (-0.43, 0.41) | 0.34 (-0.09, 0.78) | 0.48 (-0.04, 0.99) | 0.29 (-0.21, 0.80) |
| Parental Fed Up | Active Control | IBAP-BP | -0.13 (-0.61, 0.35) | -0.30 (-0.73, 0.13) | -0.28 (-0.72, 0.15) | -0.18 (-0.64, 0.29) |
| Parental Emotional Distancing | IBAP-BP | Waiting List | 0.08 (-0.41, 0.56) | 0.80 (0.34, 1.25)** | 0.74 (0.18, 1.31)** | 0.63 (0.02, 1.23)* |
| Parental Emotional Distancing | Active Control | Waiting List | -0.14 (-0.57, 0.29) | 0.54 (0.08, 1.00)* | 0.63 (0.08, 1.18)* | 0.39 (-0.18, 0.97) |
| Parental Emotional Distancing | Active Control | IBAP-BP | -0.22 (-0.73, 0.29) | -0.26 (-0.68, 0.17) | -0.12 (-0.59, 0.35) | -0.23 (-0.74, 0.27) |

| **Supplementary Table 5.**  **Comparison of Baseline Levels for Relevant Variables between those Participants who did not complete vs those who completed the Wave**  Comparison of baseline levels of parental burnout and related constructs between participants who completed versus those who did not complete follow-up assessments at 3 and 6 months. Bartlett’s test assesses homogeneity of variances, and t-tests examine mean differences. Parental burnout values are presented using the PBA average-item metric (range 0–6) rather than the raw total score. IBAP-BP = Mindfulness and Compassion-Based Inter-Care Program for Parental Burnout. **p < 0.01, *p < 0.05. Negative T values indicate that participants who answer the follow-up survey reported higher baseline levels in the variable of interest. When Bartlett’s Test was significant, Welch’s Test was used to compare groups. | | | | | | | |
| --- | --- | --- | --- | --- | --- | --- | --- |
|  |  | Baseline vs 3rd month | | Baseline vs 6th month | | Baseline vs 9th month | |
| Group | Variable | Bartlett's Test (df) | T-Test (df) | Bartlett's Test (df) | T-Test (df) | Bartlett's Test (df) | T-Test (df) |
| Full Sample | Parental Burnout | 0.03 (1) | -1.93 (591) | 0.80 (1) | -0.90 (591) | 0.04 (1) | -0.22 (591) |
| Full Sample | Parental Exhaustion | 0.65 (1) | -2.49 (591)* | 0.11 (1) | -0.84 (591) | 0.05 (1) | -0.05 (591) |
| Full Sample | Parental Identity Conflict | 0.72 (1) | -1.10 (591) | 2.19 (1) | -0.98 (591) | 0.14 (1) | -0.22 (591) |
| Full Sample | Parental Fed Up | 2.16 (1) | -2.32 (591)* | 0.88 (1) | -0.93 (591) | 0.21 (1) | -0.67 (591) |
| Full Sample | Parental Emotional Distancing | 0.01 (1) | 0.08 (591) | 0.11 (1) | -0.24 (591) | 0.01 (1) | 0.11 (591) |
| IBAP-BP | Parental Burnout | 0.19 (1) | 0.56 (159) | 0.40 (1) | 1.12 (159) | 0.05 (1) | 0.96 (159) |
| IBAP-BP | Parental Exhaustion | 0.09 (1) | 0.20 (159) | 0.34 (1) | 1.26 (159) | 0.00 (1) | 1.18 (159) |
| IBAP-BP | Parental Identity Conflict | 0.07 (1) | 0.29 (159) | 0.29 (1) | 0.51 (159) | 0.07 (1) | 0.51 (159) |
| IBAP-BP | Parental Fed Up | 0.00 (1) | 0.51 (159) | 0.04 (1) | 1.14 (159) | 0.37 (1) | 0.83 (159) |
| IBAP-BP | Parental Emotional Distancing | 0.42 (1) | 1.83 (159) | 0.12 (1) | 1.15 (159) | 0.17 (1) | 0.81 (159) |
| Active Control | Parental Burnout | 0.93 (1) | -2.31 (153)* | 1.64 (1) | -2.16 (153)* | 0.00 (1) | -2.43 (153)* |
| Active Control | Parental Exhaustion | 0.01 (1) | -1.76 (153) | 0.21 (1) | -1.82 (153) | 0.65 (1) | -2.1 (153)* |
| Active Control | Parental Identity Conflict | 2.66 (1) | -2.27 (153)* | 3.05 (1) | -2.24 (153)* | 0.8 (1) | -2.4 (153)* |
| Active Control | Parental Fed Up | 3.09 (1) | -2.80 (153)** | 3.98 (1)* | -0.25 (1490.16)* | 1.48 (1) | -2.43 (153)* |
| Active Control | Parental Emotional Distancing | 0.21 (1) | -1.20 (153) | 1.39 (1) | -1.02 (153) | 0.05 (1) | -1.42 (153) |
| Waiting List | Parental Burnout | 0.56 (1) | -1.73 (275) | 0.09 (1) | -0.68 (275) | 0.00 (1) | 0.84 (275) |
| Waiting List | Parental Exhaustion | 3.03 (1) | -2.59 (275)* | 0.35 (1) | -0.93 (275) | 0.02 (1) | 0.68 (275) |
| Waiting List | Parental Identity Conflict | 0.01 (1) | -0.18 (275) | 0.24 (1) | -0.04 (275) | 0.47 (1) | 1.39 (275) |
| Waiting List | Parental Fed Up | 0.88 (1) | -2.11 (275)* | 0.12 (1) | -0.81 (275) | 0.00 (1) | 0.07 (275) |
| Waiting List | Parental Emotional Distancing | 0.34 (1) | -0.45 (275) | 0.47 (1) | -0.49 (275) | 0.13 (1) | 0.74 (275) |

| **Supplementary Table 6.**  **ANOVA Results per Wave – Per Protocol**  Results of ANOVA tests for parental burnout and related constructs at different time points: 0 = Baseline, 1 = 3rd month, 2 = 6th month, 3 = 9th month. Levene’s test assesses homogeneity of variance, and ANOVA tests group differences. ** p < 0.01, * p < 0.05; In case Leven’s Test for variance homogeneity between groups was significant, White’s adjustment was used for heteroskedasticity-robust robust standard errors. | | | |
| --- | --- | --- | --- |
| **Variable** | **Wave** | **Levene's Test *F*(*df1, df2*)** | **ANOVA *F*(*df1, df2*)** |
| Parental Burnout | 0 | 0.25 (2, 305), p = 0.78 | 2.94 (2, 305), p = 0.05 |
| Parental Burnout | 1 | 5.8 (2, 263), p = 0.00** | 12.71 (2, 263), p = 0.00** |
| Parental Burnout | 2 | 4.13 (2, 200), p = 0.02* | 9.129(2, 200), p = 0.00** |
| Parental Burnout | 3 | 1.48 (2, 181), p = 0.23 | 5.08 (2, 181), p = 0.01* |
| Parental Emotional Distancing | 0 | 0.35 (2, 305), p = 0.70 | 2.66 (2, 305), p = 0.07 |
| Parental Emotional Distancing | 1 | 1.92 (2, 263), p = 0.00** | 12.12 (2, 263), p = 0.00** |
| Parental Emotional Distancing | 2 | 8.31 (2, 199), p = 0.00** | 6.57 (2, 199), p = 0.00** |
| Parental Emotional Distancing | 3 | 3.24 (2, 181), p = 0.04* | 4.58 (2, 181), p = 0.01* |
| Parental Exhaustion | 0 | 0.29 (2, 305), p = 0.75 | 3.02 (2, 305), p = 0.05 |
| Parental Exhaustion | 1 | 1.83 (2, 263), p = 0.16 | 1.23 (2, 263), p = 0.00** |
| Parental Exhaustion | 2 | 1.25 (2, 200), p = 0.29 | 8.3 (2, 200), p = 0.00** |
| Parental Exhaustion | 3 | .18 (2, 181), p = 0.84 | 4.54 (2, 181), p = 0.01* |
| Parental Fed Up | 0 | 1.37 (2, 305), p = 0.26 | 2.73 (2, 305), p = 0.07 |
| Parental Fed Up | 1 | 4.42 (2, 263), p = 0.01* | 8.13 (2, 263), p = 0.00** |
| Parental Fed Up | 2 | 3.5 (2, 200), p = 0.03* | 8.17 (2, 200), p = 0.00** |
| Parental Fed Up | 3 | 4.18 (2, 180), p = 0.02* | 4.04 (2, 180), p = 0.02* |
| Parental Identity Conflict | 0 | 0.42 (2, 305), p = 0.66 | 2.06 (2, 305), p = 0.13 |
| Parental Identity Conflict | 1 | 4.19 (2, 263), p = 0.02* | 10.26 (2, 263), p = 0.00** |
| Parental Identity Conflict | 2 | 4.63 (2, 200), p = 0.01* | 6.79 (2, 200), p = 0.00** |
| Parental Identity Conflict | 3 | 3.83 (2, 181), p = 0.02* | 4.72 (2, 181), p = 0.02* |

| **Supplementary Table 7.**  **Post-Hoc ANOVA Analysis to Examine Between Group Differences – Per Protocol**  Pairwise comparisons of parental burnout and related constructs across intervention groups Mindfulness and Compassion-Based Inter-Care Program for Parental Burnout (IBAP-BP), Active Control, and Waiting List at baseline, 3, 6, and 9 months. Parental burnout values are presented using the PBA average-item metric (range 0–6) rather than the raw total score. Values represent mean differences with 95% confidence intervals (CIL, CIU). Positive values indicate lower scores in the first group compared to the second. ** p < 0.01, * p < 0.05. Games-Howell test was used as a post hoc analysis as it is robust to unequal variance and unbalanced sample size between groups | | | | | | |
| --- | --- | --- | --- | --- | --- | --- |
| Variable | Group 1 | Group 2 | Baseline (CI_L_, CI_U_) | 3rd month (CI_L_, CI_U_) | 6th month (CI_L_, CI_U_) | 9th month (CI_L_, CI_U_) |
| Parental Burnout | IBAP-BP | Waiting List | 0.39 (-0.08, 0.86) | 0.93 (0.49, 1.36)** | 0.94 (0.42, 1.45)** | 0.73 (0.21, 1.26)** |
| Parental Burnout | Active Control | Waiting List | -0.09 (-0.47, 0.28) | 0.43 (0.03, 0.83)* | 0.58 (0.08, 1.08)* | 0.37 (-0.14, 0.88) |
| Parental Burnout | Active Control | IBAP-BP | -0.48 (-0.98, 0.01) | -0.50 (-0.92, -0.08)* | -0.36 (-0.83, 0.11) | -0.36 (-0.86, 0.13) |
| Parental Exhaustion | IBAP-BP | Waiting List | 0.49 (-0.04, 1.02) | 1.01 (0.48, 1.54)** | 1.06 (0.44, 1.68)** | 0.82 (0.18, 1.46)** |
| Parental Exhaustion | Active Control | Waiting List | -0.01 (-0.42, 0.40) | 0.50 (0.05, 0.95)* | 0.61 (0.04, 1.18)* | 0.47 (-0.12, 1.07) |
| Parental Exhaustion | Active Control | IBAP-BP | -0.51 (-1.06, 0.05) | -0.51 (-1.04, 0.01) | -0.45 (-1.04, 0.14) | -0.35 (-0.98, 0.29) |
| Parental Identity Conflict | Active Control | IBAP-BP | 0.18 (-0.34, 0.69) | 0.91 (0.43, 1.38)** | 0.88 (0.32, 1.45)** | 0.69 (0.15, 1.24)** |
| Parental Identity Conflict | Active Control | Waiting List | -0.27 (-0.7, 0.17) | 0.35 (-0.11, 0.81) | 0.59 (0.02, 1.15)* | 0.27 (-0.29, 0.82) |
| Parental Identity Conflict | IBAP-BP | Waiting List | -0.45 (-1.00, 0.10) | -0.55 (-1.02, -0.09)* | -0.29 (-0.80, 0.22) | -0.42 (-0.92, 0.08) |
| Parental Fed Up | IBAP-BP | Waiting List | 0.46 (-0.01, 0.94) | 0.80 (0.37, 1.23)** | 0.82 (0.34, 1.3)** | 0.61 (0.1, 1.13)* |
| Parental Fed Up | Active Control | Waiting List | -0.01 (-0.43, 0.41) | 0.34 (-0.09, 0.78) | 0.48 (-0.04, 0.99) | 0.29 (-0.21, 0.8) |
| Parental Fed Up | Active Control | IBAP-BP | -0.47 (-0.99, 0.05) | -0.46 (-0.89, -0.03)* | -0.35 (-0.80, 0.11) | -0.32 (-0.79, 0.15) |
| Parental Emotional Distancing | IBAP-BP | Waiting List | 0.04 (-0.12, 0.93) | 0.93 (0.49, 1.38)** | 0.88 (0.30, 1.46)** | 0.77 (0.16, 1.38)** |
| Parental Emotional Distancing | Active Control | Waiting List | -0.14 (-0.57, 0.29) | 0.54 (0.08, 1.00)* | 0.63 (0.08, 1.18)* | 0.39 (-0.18, 0.97) |
| Parental Emotional Distancing | Active Control | IBAP-BP | -0.54 (-1.09, 0.01) | -0.39 (-0.81, 0.02) | -0.26 (-0.74, 0.23) | -0.37 (-0.89, 0.14) |

| **Supplementary Table 8.**  **Results with Imputed data for Parental Burnout**  Waiting List Group was used as reference. Model with Random effects was used here, as no missing data was present. Random was assumed MAR for this analysis. IBAP-BP = Mindfulness and Compassion-Based Inter-Care Program for Parental Burnout. ** p < 0.01, * p < 0.05. SE = Standard Error | |
| --- | --- |
| Variable | Estimate (SE) |
| Intercept | 3.35 (0.09)** |
| Active Control | -0.06 (0.14) |
| IBAP-BP | -0.26 (0.14) |
| Wave | -0.13 (0.04)** |
| Wave X Active Control | -0.11 (0.05) |
| Wave X IBAP-BP | -0.12 (0.06)* |

| **Supplementary Table 9.**  **Multilevel Path Model for Repeated Measures – Modified Intention to Treat**  Within Level represents variations within participants, while Between Level represents variations between participants. The second column for each key variable represents the model with the interaction of group membership with time. Path model was used because it can effectively handle missing data with Full Information Maximum Likelihood (FIML). Waiting List Group was used as the reference group. IBAP-BP = Mindfulness and Compassion-Based Inter-Care Program for Parental Burnout. **p < 0.01, *p < 0.05. SE = Standard Error. | | | | | | | | | | |
| --- | --- | --- | --- | --- | --- | --- | --- | --- | --- | --- |
|  | Parental Burnout | | Parental Exhaustion | | Parental Identity Conflict | | Parental Fed Up | | Parental Emotional Distancing | |
|  | Estimate  (SE) | Estimate  (SE) | Estimate  (SE) | Estimate  (SE) | Estimate  (SE) | Estimate  (SE) | Estimate  (SE) | Estimate  (SE) | Estimate (SE) | Estimate  (SE) |
| *Within Level* |  |  |  |  |  |  |  |  |  |  |
| Wave | -0.24 (0.02)** | -0.09 (0.04)* | -0.33 (0.03)** | -0.17 (0.04)** | -0.18 (0.02)** | -0.09 (0.04)* | -0.18 (0.03)** | -0.01 (0.04) | -0.17 (0.03)** | -0.04 (0.04) |
| Wave X  IBAP-BP Group | - | -0.20 (0.06)** | - | -0.21 (0.06)** | - | -0.11 (0.06) | - | -0.24 (0.06)** | - | -0.19 (0.07)** |
| Wave X  Active Control Group | - | -0.24 (0.05)** | - | -0.26 (0.06)** | - | -0.16 (0.06)** | - | -0.28 (0.06)** | - | -0.23 (0.06)** |
|  |  |  |  |  |  |  |  |  |  |  |
| *Between Level* |  |  |  |  |  |  |  |  |  |  |
| IBAP-BP | -0.39 (0.08)** | -0.16 (0.11) | -0.44 (0.09)** | -0.19 (0.12) | -0.31 (0.08)** | -0.18 (0.11) | -0.39 (0.09)** | -0.1 (0.12) | -0.41 (0.09)** | -0.18 (0.12) |
| Active Control | -0.36 (0.08)** | -0.08 (0.10) | -0.39 (0.09)** | -0.08 (0.12) | -0.26 (0.08)** | -0.07 (0.11) | -0.37 (0.09)** | -0.04 (0.11) | -0.38 (0.08)** | -0.11 (0.11) |
| Baseline | 0.70 (0.03)** | 0.71 (0.03)** | 0.67 (0.02)** | 0.67 (0.02)** | 0.61 (0.02)** | 0.61 (0.02)** | 0.63 (0.02)** | 0.63 (0.02)** | 0.63 (0.02)** | 0.63 (0.02)** |

| **Supplementary Table 10.**  **Multilevel Path Model for Repeated Measures – Per Protocol**  Within Level represents variations within participants, while Between Level represents variations between participants. The second column for each key variable represents the model with the interaction of group membership with time. Path model was used because it can effectively handle missing data with Full Information Maximum Likelihood (FIML). Waiting List Group was used as the reference group. IBAP-BP = Mindfulness and Compassion-Based Inter-Care Program for Parental Burnout. **p < .01, *p < 0.05. SE = Standard Error. | | | | | | | | | | |
| --- | --- | --- | --- | --- | --- | --- | --- | --- | --- | --- |
|  | Parental Burnout | | Parental Exhaustion | | Parental Identity Conflict | | Parental Fed Up | | Parental Emotional Distancing | |
|  | Estimate  (SE) | Estimate  (SE) | Estimate  (SE) | Estimate  (SE) | Estimate  (SE) | Estimate  (SE) | Estimate  (SE) | Estimate  (SE) | Estimate (SE) | Estimate  (SE) |
| *Within Level* |  |  |  |  |  |  |  |  |  |  |
| Wave | -0.21 (0.02)** | -0.10 (0.04)* | -0.30 (0.03)** | -0.17 (0.04)** | -0.16 (0.03)** | -0.09 (0.04)* | -0.16 (0.03)** | -0.01 (0.04) | -0.15 (0.03)** | -0.04 (0.04) |
| Wave X  IBAP-BP Group |  | -0.12 (0.06)* |  | -0.13 (0.07) |  | -0.05 (0.07) |  | -0.18 (0.07)* |  | -0.13 (0.07) |
| Wave X  Active Control Group |  | -0.24 (0.05)** |  | -0.26 (0.06)** |  | -0.16 (0.06)** |  | -0.28 (0.06)** |  | -0.23 (0.06)** |
|  |  |  |  |  |  |  |  |  |  |  |
| *Between Level* |  |  |  |  |  |  |  |  |  |  |
| IBAP-BP | -0.41 (0.09)** | -0.27 (0.12)* | -0.46 (0.10)** | -0.32 (0.14)* | -0.33 (0.09)** | -0.28 (0.13)* | -0.47 (0.10)** | -0.26 (0.14) | -0.43 (0.10)** | -0.29 (0.14)* |
| Active Control | -0.36 (0.07)** | -0.08 (0.10) | -0.39 (0.09)** | -0.08 (0.11) | -0.27 (0.08)** | -0.07 (0.10) | -0.37 (0.08)** | -0.04 (0.11) | -0.39 (0.08)** | -0.12 (0.11) |
| Baseline | 0.70 (0.03)** | 0.70 (0.03)** | 0.67 (0.02)** | 0.67 (0.02)** | 0.61 (0.02)** | 0.61 (0.02)** | 0.63 (0.02)** | 0.64 (0.02)** | 0.63 (0.02)** | 0.63 (0.02)** |

| **Supplementary Table 11.**  **Cross-Lagged Model for Parental Risks and Resources as a Mediator.**  Cross-lagged model examining the mediating role of the Balance Between Risks and Resources (BR2)—including common and specific components—on parental burnout across time points (T0 = Baseline, T1 = 3rd month, T2 = 6th month, T3 = 9th month). The model includes estimates for the Mindfulness and Compassion-Based Inter-Care Program for Parental Burnout (IBAP-BP) and Active Control groups *compared to* the waiting list. Values represent standardized coefficients with standard errors (SE) in parentheses. The model accounts for autoregressive effects and cross-lagged relationships between BR2 and parental burnout. **p < 0.01, *p < 0.05. Fit Measures: χ^2^ = 42.81 (30) p = 0.06, CFI = 0.99, TLI = 0.98, RMSEA = 0.04 [0.00, 0.08], SRMR = 0.04 | | | | | | | | | |
| --- | --- | --- | --- | --- | --- | --- | --- | --- | --- |
|  | Parental Burnout T1 | BR2 Common T1 | BR2 Specific T1 | Parental Burnout T2 | BR2 Common T2 | BR2 Specific T2 | Parental Burnout T3 | BR2 Common T3 | BR2 Specific T3 |
| IBAP-BP | -0.58 (0.14)** | 0.5 (0.23)* | 0.52 (0.21)* |  |  |  |  |  |  |
| Active Control | -0.45 (0.13)** | 0.2 (0.22) | 0.33 (0.2) |  |  |  |  |  |  |
| Parental Burnout T0 | 0.59 (0.05)** | -0.27 (0.07)** | -0.27 (0.07)** | 0.17 (0.06)** | -0.17 (0.1) | -0.1 (0.1) | 0.01 (0.07) | 0.1 (0.12) | 0.08 (0.12) |
| BR2 Common T0 | -0.04 (0.04) | 0.47 (0.05)** |  | -0.08 (0.06) | 0.21 (0.06)** |  | 0 (0.07) | 0.25 (0.06)** |  |
| BR2 Specific T0 | -0.03 (0.05) |  | 0.46 (0.05)** | 0.04 (0.06) |  | 0.26 (0.05)** | -0.02 (0.08) |  | 0.3 (0.06)** |
|  |  |  |  |  |  |  |  |  |  |
| Parental Burnout T1 |  |  |  | 0.62 (0.07)** | -0.11 (0.12) | -0.04 (0.11) | 0.38 (0.11)** | -0.1 (0.16) | -0.06 (0.16) |
| BR2 Common T1 |  |  |  | 0.04 (0.06) | 0.45 (0.06)** |  | 0 (0.08) | 0.13 (0.07) |  |
| BR2 Specific T1 |  |  |  | -0.03 (0.07) |  | 0.46 (0.06)** | 0.02 (0.1) |  | 0.29 (0.08)** |
|  |  |  |  |  |  |  |  |  |  |
| Parental Burnout T2 |  |  |  |  |  |  | 0.36 (0.1)** | -0.17 (0.14) | -0.27 (0.15) |
| BR2 Common T2 |  |  |  |  |  |  | 0.03 (0.08) | 0.5 (0.06)** |  |
| BR2 Specific T2 |  |  |  |  |  |  | -0.07 (0.1) |  | 0.18 (0.08)* |

| **Supplementary Table 12.**  **Cross-Lagged Model for Mindfulness Facets as a Mediator.**  Cross-lagged model examining the role of mindfulness facets—Non-Judgement (NJ), Non-Reactivity (NR), Describe (DES), Awareness of the Present Moment (AWA), and Observe (OBS)—as mediators in the relationship between parental burnout across time points (T0 = Baseline, T1 = 3rd month, T2 = 6th month, T3 = 9th month). Estimates are standardized coefficients with standard errors (SE) in parentheses. The model accounts for autoregressive effects and cross-lagged relationships between mindfulness facets and parental burnout. . IBAP-BP = Mindfulness and Compassion-Based Inter-Care Program for Parental Burnout. **p < 0.01, *p < 0.05. Fit Measures: χ^2^ = 230.97 (156) p <0.01, CFI = 0.97, TLI = 0.93, RMSEA = 0.05 [0.04, 0.06], SRMR = 0.07. | | | | | | | | | | | | | | | | | | |
| --- | --- | --- | --- | --- | --- | --- | --- | --- | --- | --- | --- | --- | --- | --- | --- | --- | --- | --- |
|  | Parental Burnout  T1 | NJ  T1 | NR  T1 | DES  T1 | AWA  T1 | OBS  T1 | Parental Burnout  T2 | NJ  T2 | NR  T2 | DES  T2 | AWA  T2 | OBS  T2 | Parental Burnout  T3 | NJ  T3 | NR  T3 | DES  T3 | AWA  T3 | OBS  T3 |
| Parental Burnout T0 | 0.59 (0.05)** | -0.07 (0.04) | -0.11 (0.04)** | -0.15 (0.04)** | 0.12 (0.04)** | -0.05 (0.04) | 0.18 (0.06)** | 0.04 (0.05) | -0.02 (0.05) | -0.08 (0.05) | 0.14 (0.06)* | -0.04 (0.05) | 0.04 (0.07) | -0.01 (0.06) | 0.06 (0.06) | 0.05 (0.05) | -0.06 (0.06) | 0.06 (0.05) |
| Non Judgement T0 | 0.04 (0.06) | 0.44 (0.06)** |  |  |  |  | -0.16 (0.07)* | 0.29 (0.06)** |  |  |  |  | 0.03 (0.09) | 0.18 (0.07)** |  |  |  |  |
| Non Reactivity T0 | -0.07 (0.06) |  | 0.39 (0.05)** |  |  |  | -0.04 (0.07) |  | 0.30 (0.06)** |  |  |  | -0.03 (0.09) |  | 0.13 (0.07) |  |  |  |
| Describe T0 | 0.01 (0.07) |  |  | 0.41 (0.05)** |  |  | 0.12 (0.08) |  |  | 0.21 (0.06)** |  |  | 0.02 (0.09) |  |  | 0.25 (0.07)** |  |  |
| Awareness T0 | 0.09 (0.07) |  |  |  | 0.47 (0.06)** |  | 0.00 (0.08) |  |  |  | 0.14 (0.07) |  | -0.10 (0.10) |  |  |  | 0.11 (0.07) |  |
| Observe T0 | 0.01 (0.06) |  |  |  |  | 0.57 (0.04)** | 0.01 (0.08) |  |  |  |  | 0.27 (0.06)** | -0.02 (0.10) |  |  |  |  | 0.02 (0.06) |
| IBAP-BP | -0.59 (0.13)** | 0.15 (0.14) | 0.11 (0.13) | 0.24 (0.12)* | -0.39 (0.13)** | 0.30 (0.11)** |  |  |  |  |  |  |  |  |  |  |  |  |
| Active Control | -0.47 (0.13)** | -0.04 (0.13) | 0.23 (0.12) | 0.24 (0.11)* | -0.31 (0.12)* | 0.21 (0.11) |  |  |  |  |  |  |  |  |  |  |  |  |
| Parental Burnout T1 |  |  |  |  |  |  | 0.64 (0.07)** | -0.06 (0.06) | -0.09 (0.06) | -0.05 (0.05) | 0.02 (0.06) | -0.02 (0.05) | 0.33 (0.10)** | -0.03 (0.07) | 0.01 (0.07) | 0.03 (0.07) | 0.12 (0.07) | -0.02 (0.06) |
| Non Judgement T1 |  |  |  |  |  |  | **0.16 (0.07)*** | 0.27 (0.06)** |  |  |  |  | -0.10 (0.10) | 0.36 (0.07)** |  |  |  |  |
| Non Reactivity T1 |  |  |  |  |  |  | 0.11 (0.08) |  | 0.34 (0.06)** |  |  |  | -0.17 (0.11) |  | 0.33 (0.08)** |  |  |  |
| Describe T1 |  |  |  |  |  |  | -0.04 (0.08) |  |  | 0.35 (0.06)** |  |  | -0.22 (0.12) |  |  | 0.23 (0.07)** |  |  |
| Awareness T1 |  |  |  |  |  |  | 0.14 (0.08) |  |  |  | 0.45 (0.07)** |  | -0.16 (0.11) |  |  |  | 0.23 (0.07)** |  |
| Observe T1 |  |  |  |  |  |  | -0.05 (0.09) |  |  |  |  | 0.44 (0.06)** | **0.34 (0.14)*** |  |  |  |  | 0.20 (0.08)** |
| Parental Burnout T2 |  |  |  |  |  |  |  |  |  |  |  |  | 0.46 (0.10)** | 0.07 (0.07) | -0.14 (0.07)* | -0.13 (0.06)* | 0.12 (0.07) | -0.12 (0.06)* |
| Non Judgement T2 |  |  |  |  |  |  |  |  |  |  |  |  | 0.05 (0.11) | 0.42 (0.08)** |  |  |  |  |
| Non Reactivity T2 |  |  |  |  |  |  |  |  |  |  |  |  | 0.17 (0.11) |  | 0.27 (0.07)** |  |  |  |
| Describe T2 |  |  |  |  |  |  |  |  |  |  |  |  | 0.17 (0.14) |  |  | 0.31 (0.09)** |  |  |
| Awareness T2 |  |  |  |  |  |  |  |  |  |  |  |  | 0.07 (0.12) |  |  |  | 0.22 (0.07)** |  |
| Observe T2 |  |  |  |  |  |  |  |  |  |  |  |  | -0.22 (0.14) |  |  |  |  | 0.58 (0.08)** |

**Supplementary Table 13.**
Summary of the content and strategies of each IBAP-BP session. The table outlines the main topics addressed across the eight weekly sessions, including psychoeducational content, experiential exercises, and meditation practices. Strategies were designed to support the development of mindfulness, compassion, emotional regulation, and parenting resources. CAP refers to the "Coherent, Appeasing, and Proportional" parenting model introduced during the intervention.

| N | Session | Content | Strategies |
| --- | --- | --- | --- |
| 0 | Introduction | The model |  |
|  |  | Rules |  |
|  |  | Recommendations |  |
| 1 | Mindfulness and the Pilot | Learning, insight, and cultivating | Model presentations |
|  |  | Intention to care | Group presentation |
|  |  | Mindfulness vs automatism | Raisin meditation |
|  |  | Direct vs indirect body experience | Body scan meditation |
|  |  | Purpose | Care moment imagery and contrast exercise |
| 2 | Exploring the Mind | Wandering mind, perception and beliefs | Body scan meditation |
|  |  | Parental pressure | Week review |
|  |  | Attention, intention, and attitude | Crossing the street exercise |
|  |  | Uplifting moment | Parental pressure |
|  |  |  | Care moment imagery |
|  |  |  | Sitting meditation |
|  |  |  | Barriers and facilitators |
| 3 | The Color of Experience | Experience evaluation and its parts | Sitting meditation and care moment imagery |
|  |  | Quality time | Week review and pleasant diary |
|  |  | Scattered mind | Quality time |
|  |  | Strengths | Sound and thought meditation |
|  |  | Breathing space | Breathing space meditation and strengths |
|  |  |  | "If X, then Y" formula exercise |
| 4 | Shared Humanity | Approaching discomfort and acceptance | Movement meditation |
|  |  | Pressure–Perfectionism | Deep listening |
|  |  | CAP model of parenting | Daily discomfort review |
|  |  | Needs and shared humanity | Pressure–perfection |
|  |  |  | Mistake exercise and automatic thoughts |
|  |  |  | Perfectionism–Parenting myths |
|  |  |  | Walking meditation |
| 5 | Acceptance and Interdependence | Acceptance | Meditation with a difficult situation |
|  |  | Human needs and emotions | Boundaries situation |
|  |  | Additional breathing space | Behavior – CAP |
|  |  | Gratitude and resources | Breathing space meditation with additional steps |
|  |  | Co-parenting | Reflective listening |
|  |  |  | Needs and resources |
|  |  |  | Co-parenting |
|  |  |  | Gratitude meditation |
| 6 | Self-Compassion | Compassion and self-compassion | Self-compassion meditation |
|  |  | Compassionate communication | Boundaries |
|  |  | Co-parenting | CAP exercise |
|  |  | Asking for help | Compassionate listening |
|  |  | Care eco-resources | Co-parenting exercise |
|  |  | Co-parenting | Asking for help |
|  |  |  | Communication journal |
|  |  |  | Spiral and resources |
|  |  |  | Co-parenting |
| 7 | Compassion and Mutual Care | Care and compassion | Open meditation |
|  |  | Mutual care | Compassionate communication |
|  |  | Cultivating resources | Asking for help |
|  |  | Co-parenting | Resources journal |
|  |  |  | Co-parenting |
|  |  |  | Mutual care plan |
|  |  |  | Metta meditation |
| 8 | Cultivating the Present | Summary of information and model | Care moments imagery and Metta meditation |
|  |  | Values and principles | Group review |
|  |  | What to do moving forward | How to continue |
|  |  |  | Values and principles exercise |
|  |  |  | Closing |

**Supplementary Table 14.**

Protocol chronology, amendments, registration timeline, and deviations from the initially submitted protocol. IBAP = Mindfulness- and Compassion-Based Inter-Care Program; AC = Active Control; WL = Waiting List; PBA = Parental Burnout Assessment; FFMQ = Five Facets of Mindfulness Questionnaire; BR2 = Balance Between Risks and Resources; AE = Adverse Effects; mITT = Modified Intention-to-Treat; SEM = Structural Equation Modeling; DID = Difference-in-Differences; LGM = Latent Growth Model; SAP = Statistical Analysis Plan; N/P = Not provided / not prespecified.

| Domain | Original protocol | Final implementation | Date | Timing | Notes |
| --- | --- | --- | --- | --- | --- |
| Protocol approval | Ethics approval | — | Oct 27, 2022 | Pre-enrollment | Original protocol approved |
| Study arms | 2 arms (IBAP vs AC) | 3 arms (IBAP, AC, WL) | 20 Dec 2022 | Pre-enrollment | Ethics-approved amendment |
| Sample size | 212–250 planned | 593 randomized | Pre + during recruitment | Pre + during | Expanded (logistics + WL replacement) |
| Enrollment period | Planned | Dec 22, 2022 – Mar 8, 2023 | N/P | N/P | As planned |
| Trial registration | Not registered | Registered (NCT05833269) | Apr 15, 2023 | Post-enrollment start | Retrospective registration |
| Registry (initial) | 2 arms (IBAP, AC) | — | Apr 15, 2023 | Post-enrollment start | WL omitted (admin error) |
| Registry update (WL) | — | 3 arms (IBAP, AC, WL) | Aug 15, 2023 | Post-enrollment | WL added; reflects protocol |
| Primary outcome | PBA (9 months) | Unchanged | N/P | Pre-enrollment | Prespecified |
| Secondary outcomes | FFMQ, BR2, AE | Unchanged | N/P | Pre-enrollment | Prespecified |
| Additional outcomes | N/P | Microbiota substudy | Jan 31, 2023 | During enrollment | Ethics-approved; separate report |
| Statistical plan | ANOVA, t-test, longitudinal models (e.g., DID, LGM) | + mITT, SEM, mediation, subgroup, imputation | N/P | Pre + post | Core prespecified; extensions exploratory |
| Mediation / SEM | N/P | SEM, cross-lag models | N/P | Post-enrollment | Exploratory |
| SAP registration | Not preregistered | Defined in manuscript | N/P | N/P | No formal SAP; transparent reporting |

**References**

1. Manrique-Millones D, Vasin GM, Dominguez-Lara S, et al. Parental Burnout Assessment (PBA) in Different Hispanic Countries: An Exploratory Structural Equation Modeling Approach. *Frontiers in Psychology*. 2022;13. Accessed July 7, 2022. https://www.frontiersin.org/articles/10.3389/fpsyg.2022.827014

2. Villalón, Francisco, Escaffi-Schwarz M, Mundt A. Validación de la escala Five Facet Mindfulness Questionnaire en estudiantes de medicina y médicos en Chile. *Rev Med Chile*. 2023;151:435-445.

3. Mikolajczak M, Roskam I. A Theoretical and Clinical Framework for Parental Burnout: The Balance Between Risks and Resources (BR2). *Front Psychol*. 2018;9:886. doi:10.3389/fpsyg.2018.00886

4. Cebolla A, Demarzo M, Martins P, Soler J, Garcia-Campayo J. Unwanted effects: Is there a negative side of meditation? A multicentre survey. *PLOS ONE*. 2017;12(9):e0183137. doi:10.1371/journal.pone.0183137

5. Olivares-Faúndez V, Mena-Miranda L, Macía-Sepúlveda F, Jélvez-Wilke C. Validez factorial del Maslach Burnout Inventory Human Services (MBI-HSS) en profesionales chilenos. *Universitas Psychologica*. 2014;13(1). doi:10.11144/Javeriana.UPSY13-1.vfmb

6. Juyumaya JE. ESCALA UTRECHT DE WORK ENGAGEMENT EN CHILE: MEDICIÓN, CONFIABILIDAD Y VALIDEZ. 2019;1:16.

7. Soler J, Franquesa A, Feliu-Soler A, et al. Assessing Decentering: Validation, Psychometric Properties, and Clinical Usefulness of the Experiences Questionnaire in a Spanish Sample. *Behavior Therapy*. 2014;45(6):863-871. doi:10.1016/j.beth.2014.05.004

8. Cabello R, Salguero JM, Fernández-Berrocal P, Gross JJ. A Spanish Adaptation of the Emotion Regulation Questionnaire. *European Journal of Psychological Assessment*. 2013;29(4):234-240. doi:10.1027/1015-5759/a000150

9. Madrid HP, Vasquez CA, Patterson M. Measurement of the Psychosocial Work Environment in Spanish: Validation of the Psychosocial Factors Questionnaire 75 (PSF-Q75) to Capture Demands and Resources at Different Levels of Analysis. *Front Psychol*. 2020;11:580196. doi:10.3389/fpsyg.2020.580196

10. Ortet G, Mezquita L, Morizot J, Ortet-Walker J, Ibáñez MI. Assessment of “los pequeños” Big Five: The Spanish version of the Big Five Personality Trait Short Questionnaire in adolescents. *Psychol Assess*. 2022;34(5):e32-e44. doi:10.1037/pas0001119

11. Lee S, Lee DK. What is the proper way to apply the multiple comparison test? *Korean J Anesthesiol*. 2018;71(5):353-360. doi:10.4097/kja.d.18.00242

12. Kline RB. *Principles and Practice of Structural Equation Modeling*. 4th ed. The Guilford Press; 2015.

13. Schermelleh-Engel K, Moosbrugger H, Müller H. Evaluating the Fit of Structural Equation Models: Tests of Significance and Descriptive Goodness-of-Fit Measures. *Methods of Psychological Research Online*. 2003;8(2).

14. Little TD. *Longitudinal Structural Equation Modeling*. The Guilford Press; 2013.

15. MacKinnon DP, Krull JL, Lockwood CM. Equivalence of the Mediation, Confounding and Suppression Effect. *Prevention Science*. 2000;1(4):173-181. doi:10.1023/A:1026595011371

16. Singer JD, Willet BJ. *Applied Longitudinal Data Analysis*. Vol 6. Oxford University Press; 2003. doi:10.1093/acprof
